# Selective decision-making and collective behavior of fish by the motion of visual attention

**DOI:** 10.1093/pnasnexus/pgae264

**Published:** 2024-07-02

**Authors:** Susumu Ito, Nariya Uchida

**Affiliations:** Department of Physics, Tohoku University, Sendai 980-8578, Japan; Department of Physics, Tohoku University, Sendai 980-8578, Japan

**Keywords:** fish schooling, visual information, agent-based model, decision-making

## Abstract

Collective motion provides a spectacular example of self-organization in Nature. Visual information plays a crucial role among various types of information in determining interactions. Recently, experiments have revealed that organisms such as fish and insects selectively utilize a portion, rather than the entirety, of visual information. Here, focusing on fish, we propose an agent-based model where the direction of attention is guided by visual stimuli received from the images of nearby fish. Our model reproduces a branching phenomenon where a fish selectively follows a specific individual as the distance between two or three nearby fish increases. Furthermore, our model replicates various patterns of collective motion in a group of agents, such as vortex, polarized school, swarm, and turning. We also discuss the topological nature of the visual interaction, as well as the positional distribution of nearby fish and the map of pairwise and three-body interactions induced by them. Through a comprehensive comparison with existing experimental results, we clarify the roles of visual interactions and issues to be resolved by other forms of interactions.

**Significance Statement**

In the movement of various organisms, decision-making through visual information is considered to play a crucial role. Here we focus on fish to elucidate the impact of selective decision-making on their coordinated motion. Based on experimentally observed behaviors of fish, we propose an agent model equipped with a visual system. The model describes selective decision-making through the movement of direction of visual attention, providing a comprehensive explanation for the interactions of a few fish and patterns of collective motion.

## Introduction

Collective motion and cluster formation are ubiquitously found in various organisms (1). Fish are no exception: there is swarm in which the direction of motion of fish is random, polarized school which shows directional movement, and vortex in which fish rotates around an axis (2–5). Schooling is induced not only by hydrodynamic interactions (6–9), but also for efficiently foraging food (10) and avoiding predator (2, 11), in which visual cues play an important role in transferring information among individuals (10, 12–15).

How does a fish process visual information in a school? Reacting to all neighboring fish within the field of view imposes a heavy load on the information processing system (16), and it is considered to be less suitable for fish with relatively small brains (15, 17). In fact, zebrafish (*Danio rerio*) show selective decision making (18, 19): when there are two or three targets (i.e. light spots or virtual fish) within a certain distance in the range of eye sight, a zebrafish is attracted to one of the targets. In other words, a zebrafish selects and pays attention to one target, by truncating the visual information from other targets. A similar behavior is observed for fly and locust (19), indicating the commonness of selective decision-making through visual information.

Fish schools have been modeled by agent-based models for decades (20–24) with simple geometric criteria to determine the interaction: metric range (25–27), topological distance (28–31), and Voronoi tessellation (7, 32–36). Recent studies incorporate visual information for modeling the collective motion of fish (37), bird (38), and generic agents (39–43). These models assume that an agent can integrate the information (e.g. distance and velocity) of all neighbors detected to decide its action. Some models (19, 44–46) treat selective decision-making under specific conditions. Collignon *et al.* (44) used a stochastic model to reproduce the motion of 10 fish exhibiting burst-coast swimming in a tank with feeders. Sridhar *et al.* (19) and the successive studies (45, 46) introduced a spin model inspired by a cooperative phenomena in neural networks. This model was applied to an agent interacting with a few virtual agents, and explained a bifurcation behavior of the agent’s position with the change of the distance between the targets. However, it remains elusive whether selective decision-making via visual interactions can explain the collective behavior of real fish.

In this paper, we propose a comprehensive model of selective decision-making and resultant collective motion based on visual stimuli. We introduce the notion of direction of attention induced by the stimulus and model its movement induced by the visual stimuli from neighbor fish. The fish interact via a repulsive, attractive and alignment interactions with the neighbors in the direction of attention. For a few fish, our model reproduces the bifurcation behavior observed in the previous experiment (19). For many fish, our model exhibits various patterns of emergent collective motion (vortex, polarized school, swarm, and turning). Furthermore, we analyze the topological distance (17) and the pairwise and three-body forces (47) from the viewpoint of visual interactions. The roles of visual information in the movement of fish is discussed by comprehensive comparison with the experimental results.

## Experimental background

Our model is based on the visual system and experimentally observed behaviors of fish. Here, we explain the experimental background of the model.

First, we consider the mechanism of the eye and the transmission of information. Each fish detects its neighbors as projected images on the retina. The signal is created by the photoreceptor cells and is transmitted to the visual centers of the brain through retinal ganglion cells (48). The photoreceptor cells and a ganglion cell have a many-to-one coupling, and therefore the signal at a ganglion cell contains the information of the spatial width of an image (49). Moreover, the signals from ganglion cells are transmitted to the visual cortex and create a map called the retinotopic map, which preserves the position on the retina (50).

The resolution of the images are determined by the number of ganglion cells. Their density is on the order of 10^4^ cells/mm^2^ for zebrafish and golden shiner (*Notemigonus crysoleucas*), which use visual information as primary source of information. In addition, the resolution is higher in the forward direction than the backward: the density of the ganglion cells is higher in the rear side of the retina than in the front (48).

Next, the visual stimuli contain voluminous information, from which fish select specific information to determine their motion. An experiment on larval zebrafish shows that the visual interaction mainly depends on the vertical size of the image, and starts to depend weakly on the horizontal size as they grow (51). It is considered that fish adopts the vertical size as visual information to precisely measure the distance to neighbors, because the horizontal size easily changes by the relative heading angle of the neighbor as the fish has a slender body (51).

The eyeball of fish moves well (52), and fish can track moving targets (53). The motion velocity of an image as well as its size are encoded on the brain (15): the macroscopic spatial pattern of the targets (54) and its velocity (55) determine the intensity of a signal via the neural system including the ganglion cells. An experiment using golden shiner shows that a fish tends to follow the neighbors at a close distance in the front, as well as those moving at high relative speed. (56). Therefore, we incorporate the relative position and speed of the neighbor agents as the key elements for the visual interaction.

Finally, we describe the kinematic reaction to the stimuli. The interaction forces are classified into repulsion, attraction, and alignment. Repulsion at short distances and attraction at large distances are demonstrated by a force map for golden shiner (47) and mosquitofish (*Gambusia holbrooki*) (17). The aligning interaction is found later for rummy-nose tetra (*Hemigrammus rhodostomus*) (57). It is also worth mentioning that the alignment of zebrafish is not explained by repulsion and attraction only (51). Integrating these properties, we construct our model as follows.

## Model

First, we present the overall construction of the model. A self-propelled agent (fish) perceives the visual stimuli from neighbors, and the visual system embedded in each agent produces signals containing information on the spatial width of an image. Each signal whose intensity based on the vertical size and the velocity of the neighbor is transferred to the brain system and synthesized as the retinotopic map. The visual attention moves to the strong signal on the map. An agent reads the visual information (e.g. the position and the relative orientation) of the neighbor on the line of visual attention, and then determines its own motion based on the kinematic reaction rule.

**Fig. 1.**
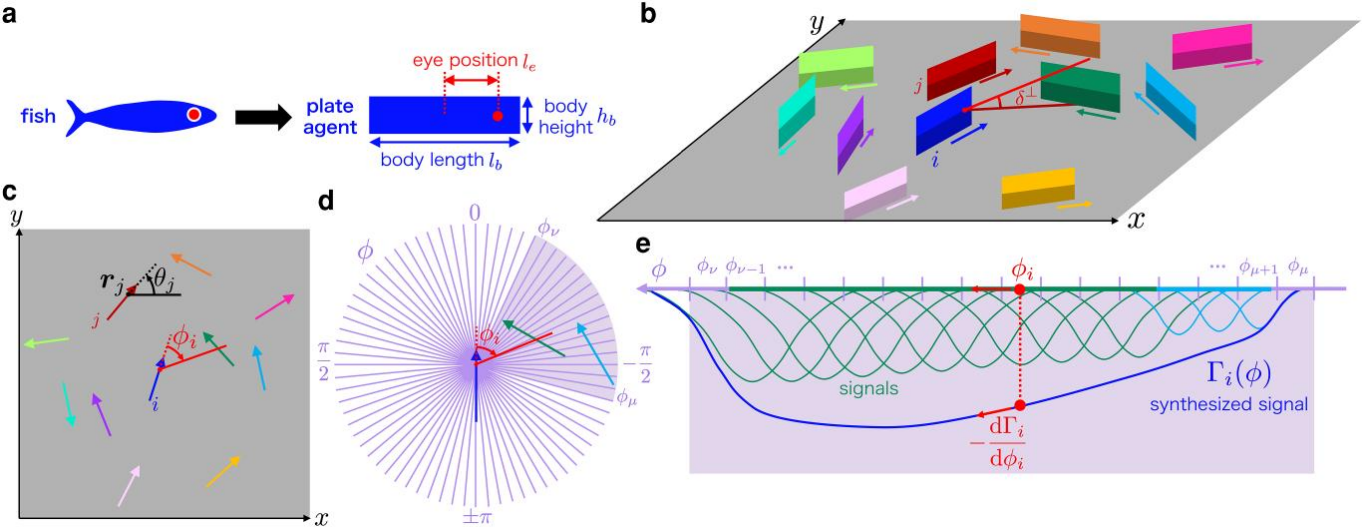
Schematic illustration of our model. a) An agent is a rectangular plate of length $l_{b}$ and height $h_{b}$. The eye is positioned at distance $l_{e}$ from the body center. b) The agents move in a *x*-*y* plane. Each arrow represents the direction of motion, and $\delta^{⊥}$ is the vertical angular diameter of the green agent measured by the eye of the blue agent (*i*). It varies over the body of the green agent. c) The top view of b). The angle $\phi_{i}$ gives the direction of visual attention of *i* relative to the head direction. $r_{j}=(x_{j},y_{j})$ and $\theta_{j}$ are the eye position and the angle of orientation of *j*. Both of the angles are measured in the counterclockwise direction and take values in [−π,π]. d) The visual field of the blue agent (i) is a circle parametrized by the angle ϕ∈[−π,π] and separated into bins. The two neighbors (green and cyan) are detected in the bins in $\phi_{\mu }\leq \phi \leq \phi_{\nu }$. e) The signals from the images in $\left[\phi_{\mu },\phi_{\nu }\right]$ in d). The green and cyan lines on the *ϕ* axis show the bins in the visual field occupied by each agent. The green and cyan curves show the signals $\gamma_{i,\lambda }(\phi )$ from the *λ*th bin that are superimposed as the synthesized signal $\Gamma_{i}(\phi )$ in the perception field. The red arrow shows the derivative $-\text{d}\Gamma_{i}/\text{d}\phi_{i}$.

Next, we show the details of the model. We model each self-propelled agent as a rectangular plate of length $l_{b}$, height $h_{b}$ which has a monoeye at distance $l_{e}$ its center (Fig. 1a), and consider *N* agents moving on a plane with no boundary (Fig. 1b). For the agent indexed by i=1,2,…,N, we define the position of the eye $r_{i}=(x_{i},y_{i})$ and the velocity $v_{i}=\text{d}r_{i}/\text{d}t=v_{i}e_{i}$, where $v_{i}\geq 0$ is the speed, and $e_{i}=(\cos\;\theta_{i},\sin\;\theta_{i})$ as the unit vector in the direction of motion ($\theta_{i}$ is the angle of orientation).

The essential new feature of our model is the direction of visual attention specified by its angle $\phi_{i}$ (see Fig. 1c). We model the motion of $\phi_{i}$ as follows. As shown in Fig. 1d, we divide the visual field into bins, each of which corresponds to a ganglion cell. The number of bins $N_{b}$ corresponds to the number of the ganglion cells which is estimated by the density of the cells and the size of an eye. The angle of the *μ*th bin ($\mu =1,2,\ldots ,N_{b}$) is denoted by $\phi_{\mu }$. The vertical angular diameter $\delta_{i,\mu }^{⊥}$ (see Fig. 1b) and the relative speed $u_{i,\mu }$ of the *i*th neighbor in a bin *μ* are encoded as the visual information. The dependences on the vertical angular diameter and the relative speed of the signal are described by $A(\delta_{i,\mu }^{⊥})$ and $U(\delta_{i,\mu }^{⊥},u_{i,\mu };\beta )$, respectively. The function *A* increases with the vertical angular diameter (and therefore decreases with the distance), and *U* increases with the relative speed of the neighbor. The function *U* also depends on the vertical angular diameter and is large in a distance where the pattern on the body of the neighbor can be detected. (See *Materials and Methods* for details.)

We represent the retinotopic map on the visual cortex by the one-dimensional “perception field” with the angular coordinate ϕ∈[−π,π]. The signal from each ganglion cells has a distribution centered at $\phi_{\mu }$ on the perception field (see a signal in Fig. 1e):


(1) $$\gamma_{i,\mu }(\phi )=-U(\delta_{i,\mu }^{⊥},u_{i,\mu };\beta )A(\delta_{i,\mu }^{⊥})G(\phi ,\phi_{\mu };\kappa ).$$

The angle dependence is given by the periodic function of *ϕ*,


(2) $$G(\phi ,\phi_{\mu };\kappa )=\text{exp}[\kappa \{\cos\;(\phi -\phi_{\mu })-1\}],$$

which is the signal with the unit intensity: G=1 at the center $\phi =\phi_{\mu }$. κ>0 gives the sharpness of a signal. When *κ* is large, the width of the signal becomes narrow and the accuracy of the location of the visual information source increases.

The signals from many ganglion cells are superimposed as the synthesized signal (see Fig. 1e):


(3) $$\Gamma_{i}(\phi )=\sum_{\mu =1}^{N_{b}}D(\phi_{\mu };\chi )\gamma_{i,\mu }(\phi ),$$

where the function $D(\phi_{\mu };\chi )$ represents the density of ganglion cells and has the aforementioned front–back asymmetry parametrized by *χ*. For large *χ*, the amplitude of the signal located in the front of the fish relatively increases because the front is more clearly visible than the back.

The angle of direction of attention $\phi_{i}$ is driven by the equation of motion


(4) $$\tau_{\phi }\frac{\text{d}\phi_{i}}{\text{d}t}=-\frac{\text{d}\Gamma_{i}(\phi_{i})}{\text{d}\phi_{i}},$$

where $\tau_{\phi }$ is the characteristic time scale for $\phi_{i}$. The derivative $-\text{d}\Gamma_{i}(\phi_{i})/\text{d}\phi_{i}$ represents the gradient force for the visual attention (see Fig. 1e). In other words, $\phi_{i}$ tends to approach a the local minimum of $\Gamma_{i}(\phi )$ which is regarded as a potential function in the perception field. The local minimum is deeper when the neighbor is closer, and/or is in the front, and/or has a higher relative speed which are the important visual informations that fish select.

Next, we introduce the equation of motion of the position of the *i*th agent. We adopt the variables ($v_{i},\theta_{i}$) for the equation of motion (41, 42) because, in addition to the change of the orientation, the change of the speed is also important for fish (17, 47). The equation for $v_{i}$ is


(5) $$\frac{\text{d}v_{i}}{\text{d}t}=C(v_{0}^{2}-v_{i}^{2})+\langle F(\phi_{\mu },\delta_{i,\mu }^{⊥})\rangle_{\mu }+\eta_{v,i},$$

and the equation for $\theta_{i}$ is


(6) $$\frac{\text{d}\theta_{i}}{\text{d}t}=\langle \Omega (\phi_{\mu },\delta_{i,\mu }^{⊥},\psi_{i,\mu })\rangle_{\mu }+\eta_{\theta ,i},$$

where the mass is normalized as one, $v_{0}$ is the steady swimming speed, and *C* is a constant. The self-propelled term $C(v_{0}^{2}-v_{i}^{2})$ corresponds to the balance between Newton’s drag force $-Cv_{i}^{2}$ and the thrust force $Cv_{0}^{2}$ (58). The effect of the visual attention $\phi_{i}$ is included in the interaction terms: the speeding force term $\langle F(\phi_{\mu },\delta_{i,\mu }^{⊥})\rangle_{\mu }$ and the angular velocity term $\langle \Omega (\phi_{\mu },\delta_{i,\mu }^{⊥},\psi_{i,\mu })\rangle_{\mu }$. Here $\langle \cdots \rangle_{\mu }$ means averaging over the neighbors in the line of visual attention ($\phi_{i}$). To be precise, we take the neighbors in five bins that are centered around $\phi_{i}$ and has the width of horizontal resolution angular diameter $\delta_{0}^{∥}$. In other words, it corresponds to reading the information on the line of visual attention within the resolution range. Here, we explain the qualitative meanings of the interaction functions based on experimental kinematic evidences. The speeding force $F(\phi_{\mu },\delta_{i,\mu }^{⊥})$ depends on the orientational position $\phi_{\mu }$ and the vertical angular diameter $\delta_{i,\mu }^{⊥}$ of the neighbor. The repulsion acts at a far region ($\delta_{i,\mu }^{⊥}$ is small) and the attraction acts at a near region ($\delta_{i,\mu }^{⊥}$ is large), and strong force acts in the front and rear ($\phi_{\mu }\approx 0,\pm \pi$) (17, 47). The angular velocity $\Omega (\phi_{\mu },\delta_{i,\mu }^{⊥},\psi_{i,\mu })$ depends on $\phi_{\mu },\;\delta_{i,\mu }^{⊥}$, and $\psi_{i,\mu }$ which is the relative heading angle between the focal agent and the neighbor detected at $\phi_{\mu }$. The angular velocity is composed of the repulsion–attraction and the alignment terms (57). The repulsion–attraction is strong on the left and right ($\phi_{\mu }\approx \pm \pi /2$) (17, 47). It is also strong when the neighbor moves in the opposite direction ($\psi_{i,\mu }\approx \pm \pi$) (57) inside the distance where the pattern of the neighbor is visible. On the other hand, the alignment is strong in the front ($\phi_{\mu }\approx 0$) and when the neighbor moves perpendicularly ($\psi_{i,\mu }\approx \pm \pi /2$) at the medium distance (57). Finally, $\eta_{v,i}\text{d}t=\sqrt{2D_{v}}\text{d}w_{v,i}$ and $\eta_{\theta ,i}\text{d}t=\sqrt{2D_{\theta }}\text{d}w_{\theta ,i}$ correspond to the white Gaussian noises where $\text{d}w_{v,i}$ and $\text{d}w_{\theta ,i}$ are the standard Wiener processes.

In our model, we rescaled the parameter values for nondimensionalization by a body length (BL) $l_{b}$, timescale 1 sec, and body mass. (For example, the case in which the speed takes 1 means that the speed is 1 BL/sec.) The main control parameters of our model are the heterogeneity of the density of ganglion cells *χ* and the strength of alignment $\omega_{o}$. (See *Materials and Methods* and supplementary material Text for details of the formulation and definitions of the quantities measured in the simulation.)

## Results

### Behavior of selective decision-making

**Fig. 2.**
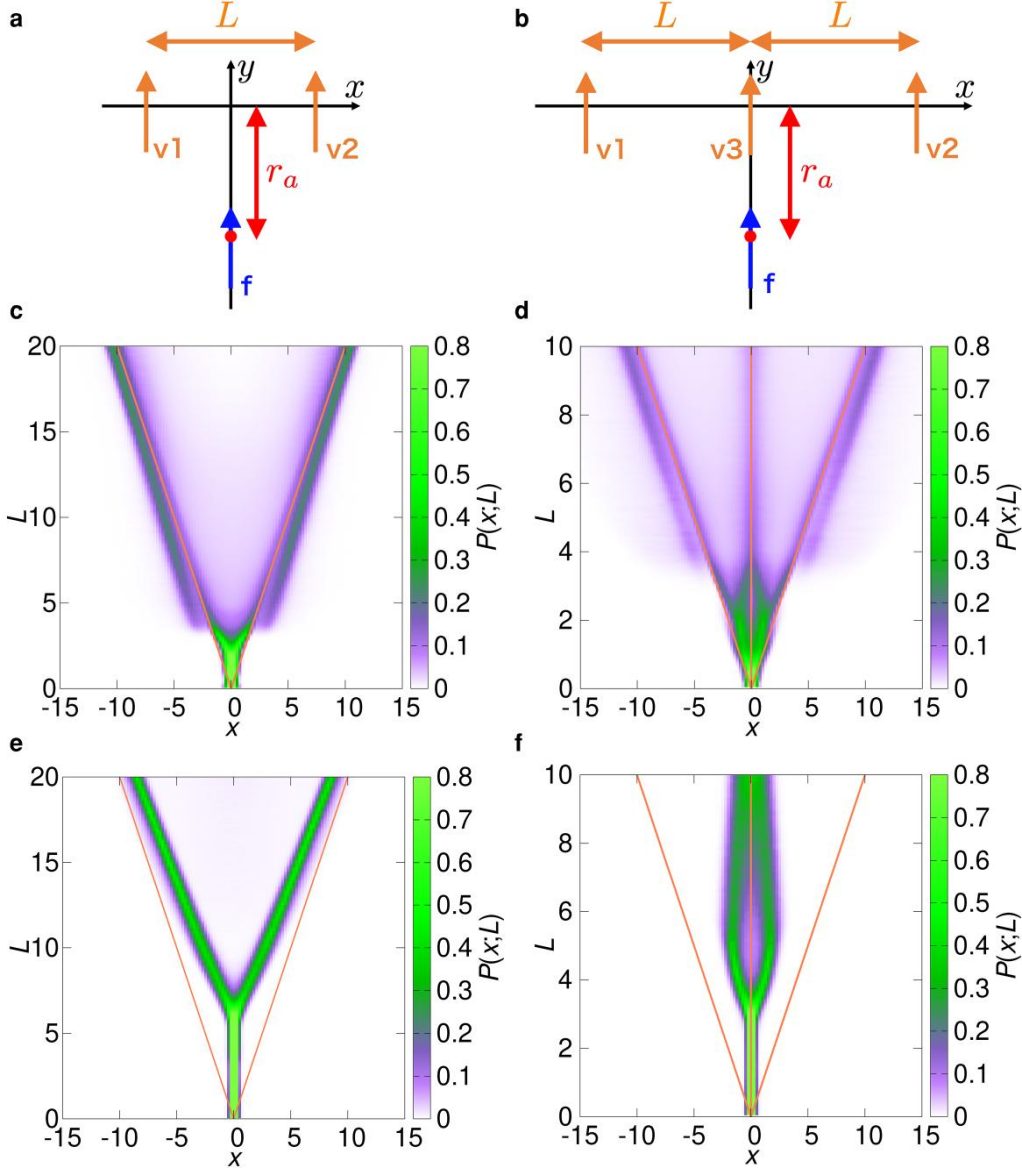
Selective decision-making for a,c,e) two and b,d,f) three virtual agents with $\chi =0.3,\omega_{o}=1.0$. a)–b) Schematic illustration of initial condition. A blue arrow is a focal agent f which will move freely in *x*-*y* plane, and the orange arrows are the virtual agents vn which will move along *y*-axis. The marginal probability distribution P(x;L) of c,d) our model and e,f) the conventional particle model. The orange lines represent the *x* position of virtual agents.

First, we demonstrate that our model exhibits selective decision-making by reproducing some key features of the experimental results in (19). Following the study, we consider a focal agent following a few “virtual agents” that perform prescribed motion without interacting with the other agents. In the initial state (t=0), as shown in Fig. 2a–b, the focal agent f located at $x_{f}=0$, $y_{f}=-r_{a}$ is moving in the *y*-direction with the speed $v_{f}=v_{0}$ and the angle of visual attention $\phi_{f}=0$. The virtual agents vn (n=1,2,3) are located at $y_{\mathrm{vn}}=0$ with the lateral distance *L* and move along the *y*-axis with the speed $v_{\mathrm{vn}}=v_{0}$. The focal agent f obeys the time-evolution Eqs. 4–6 and follows the virtual agents, which maintain their relative distance and velocity in the *y*-direction. Here we set the parameters as χ=0.3 and $\omega_{o}=1.0$.

The focal agent fluctuates between different agents exhibiting a back-and-forth motion (Fig. S3a,d), which has a characteristic timescale on the order of 10 time unit. Trajectories plotted in the moving frame of the virtual agents show that the focal agent follows the virtual agents from the back (Fig. S3b,e).

To study the dependence on the lateral distance *L*, we use the positional distribution of the focal agent P(x,y;L) in the moving frame and the marginal probability distribution P(x;L) which is obtained by integrating P(x,y;L) over the *y*-axis. The plots of P(x,y;L) in Figs. S4, S5 show that the focal agent tends to be located behind the center of the virtual agents for a small *L*, while the peak is split and located just behind each virtual agent for larger values of *L*. This bifurcation behavior is more clearly seen in the plots of P(x;L) in Fig. 2c,d, which resemble the experimental results for fish (19). For two virtual agents (Fig. 2c), the focal agent is located at the center of the virtual agents for L≲3, and at the position of the virtual agents for L≳3. For three virtual agents (Fig. 2d), P(x;L) shows a three-way fork through two consecutive bifurcations; for L≲1, the focal agent is at the center of the three virtual agents; for 1≲L≲3, it resides at the center of v1 and v3 or the center of v2 and v3; for L≳3, it comes to the position of the virtual agents. At a very large distance (L∼10), the probability to follow the center virtual agent v3 becomes smaller than those for v1 and v2. See supplementary material Text and Figs. S6, S67 for dependence on the other parameters.

We also tested an asymmetrical configuration of three virtual agents, where v3 is shifted to the right by the distance $L_{\mathrm{asym}}$ from the center (see Fig. S8a). For intermediate values of $L_{\mathrm{asym}}$, the marginal probability distribution exhibits three peaks at the start of the three-way fork bifurcation (L=3.0), instead of the four peaks for $L_{\mathrm{asym}}=0$. The peaks are located at the right of v1, the left of v3, and between v2 and v3 (see Fig. S8g).

The behaviors are compared with those of a conventional particle model, which determines the motion of the focal agent by averaging the forces exerted by all virtual agents. For two virtual agents, we observed a bifurcation as shown in Fig. 2e, but the distribution P(x;L) is determined by the equilibrium position of the averaged forces. In fact, the focal agent in the conventional particle model stays around a certain position between virtual agents instead of showing a back-and-forth motion (see Fig. S3c,f). For three virtual agents, P(x;L) shows a two-way fork pattern that closes at large *L* as shown in Fig. 2f, instead of a three-way fork. The focal agent does not select and approach the left and right virtual agents.

### Collective motion

Next we study collective motion of many agents. For the initial condition, we randomly positioned 100 agents in a circle with a radius of 7 length unit. They have the same initial speed $v_{i}=v_{0}$ with the moving directions $\theta_{i}$ and angle of visual attention $\phi_{i}$ uniformly distributed in [−π,π].

**Fig. 3.**
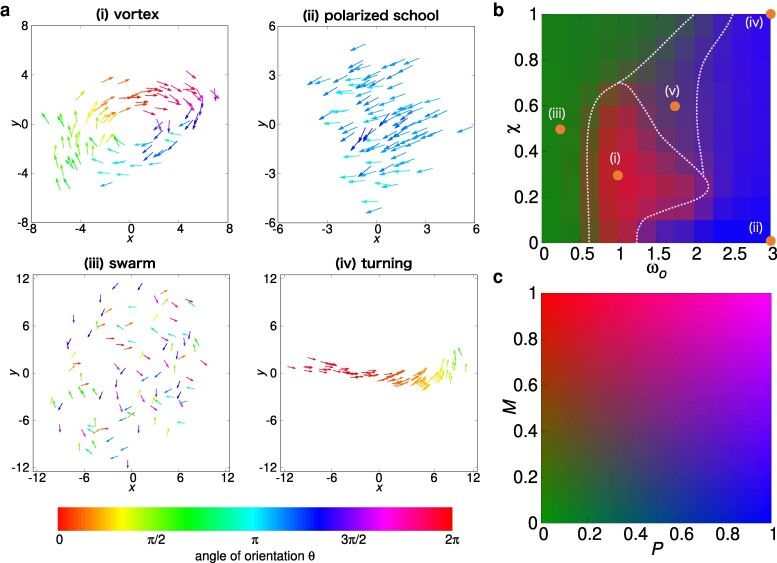
The snapshots of cluster and the phase diagram of pattern. (i)–(v) correspond to the pattern and the parameters $\left(\chi ,\omega_{o}\right)$ as follows. (i) vortex: $\chi =0.3,\omega_{o}=1.0$. (ii) polarized school: $\chi =0,\omega_{o}=3.0$. (iii) swarm: $\chi =0.5,\omega_{o}=0.25$. (iv) turning: $\chi =1.0,\omega_{o}=3.0$. (v) unsteady aggregation: $\chi =0.6,\omega_{o}=1.75$. a) The snapshots of 100 agents which are represented as arrows of a body length $l_{b}$. The color corresponds to the direction of motion. b) The phase diagram with respect to $\left(\chi ,\omega_{o}\right)$. The color represents the value of order parameters (P,M) as shown in (c). The orange points correspond to each parameter $\left(\chi ,\omega_{o}\right)$ of (i)–(v). The dashed lines represent the qualitative boundary of the patterns.

Varying the parameters $\left(\chi ,\omega_{o}\right)$, we obtained the collective patterns shown in Figs. 3a, S9. They are classified into (i) vortex: agents rotating around a common axis, (ii) polarized school: agents aligned and moving coherently in the same direction, (iii) swarm: randomly oriented agents, (iv) turning: an elongated and curved cluster that intermittently develops from a polarized school (see also Fig. S9a), and (v) unsteady aggregation: a vortex collapses and then is transformed into a polarized school, which becomes a vortex again. This cycle occurs repeatedly with irregular time intervals (see Fig. S9b). See Supplementary material Movies S1–S5 for the dynamics of (i)–(v), respectively. The size of these patterns read from Fig. 3 is on the order of 10 length unit. The parameters $\chi =0.3,\omega_{o}=1.0$ that were used to study selective decision making in the previous section corresponds to the vortex pattern.

As quantitative measures of the patterns, we introduce the polar order parameter *P* and the rotational order parameter *M* (see *Materials and Methods*). For the steady patterns (i)–(iii), the order parameters rapidly converge to constants (see Fig. S10). For (iv), both P(t) and M(t) exhibit spikes that correspond to the emergence of curved clusters from a polarized school. For (v), both P(t) and M(t) oscillate with large amplitudes. For each parameter set ($\chi ,\omega_{o}$), we performed 25 simulations in the time domain t∈[0,800] to obtain the average values of *P* and *M*. The plot of *P* and *M* in the ranges χ∈[0,1], $\omega_{o}\in [0,3]$ is shown in Fig. 3b. We consider the noiseless case ($D_{v}=D_{\theta }=0$) to maximize the stability of the clusters. The plot gives a phase diagram of the collective patterns. As the strength of alignment $\omega_{o}$ increases, the agents get aligned and the cluster changes from a swarm to a vortex, and then to a polarized school or turning. For χ≲0.5, the vortex emerges for $\omega_{o}≲1.5-2.0$, and the polarized school for $\omega_{o}≳1.5-2.0$. For χ≳0.5, we obtain unsteady aggregation for $1.5≲\omega_{o}≲2.0$, and turning for $\omega_{o}≳2.0$. Note that the emergence of swarm is controlled by $\omega_{o}$ and is almost independent of *χ*. See also Fig. S11 for a phase diagram based on other quantities.

The dependence on the number of agents *N* is studied by keeping the same number density in the initial condition. As shown in Fig. S15a,b, the cluster tends to be stable for 50≲N≲200, but splits frequently for both 4≲N≲50 and N≳200. The splitting is a complex dynamical process: a large cluster with N≳200 splits into several small clusters. A resultant cluster with 50≲N≲200 remains stable, but a small fragment with 4≲N≲50 is further splitted. A smaller cluster with a few number of agents is captured in a relatively large cluster by attraction, and that large cluster may split or remain stable. It is difficult to obtain statistical information for the chaotic time series. (See supplementary material Text and Figs. S12, S14 for a detailed analysis of the splitting and its dependence on the other parameters.)

### Visual information and topological distance

**Fig. 4.**
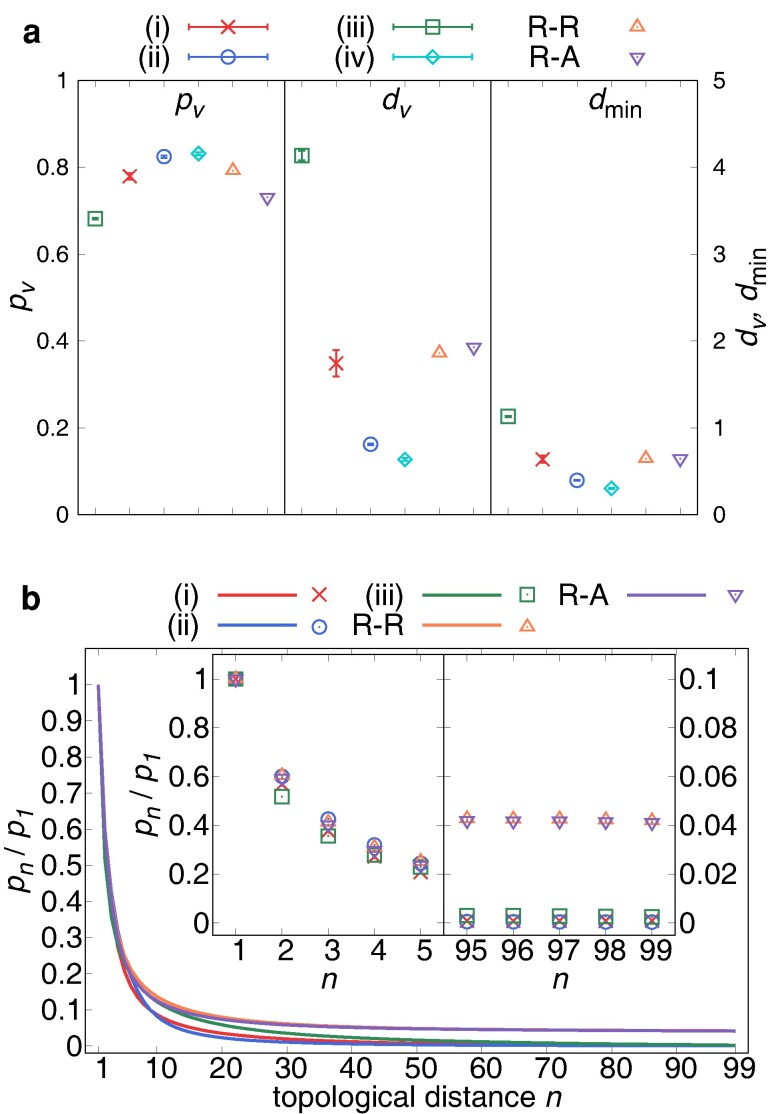
Visual information of an agent in a (i) vortex (red) (ii) polarized school (blue), (iii) swarm (green), and (iv) turning cluster (cyan). R-R (orange) and R-A (purple) are the abbreviations of the random-random case and the random-aligned case (see text). a) The occupancy ratio $p_{v}$, the average distance $d_{v}$ and the minimum distance $d_{\min}$ to the neighbors. The error bar represents the standard deviation. b) The relative occupancy ratio $p_{n}$ normalized by $p_{1}$. Insets show the enlarged figure for the topological distance n∈[0,5] and n∈[95,99].

Here, we analyze the visual information of an agent in a cluster with 100 agents. We measure the occupancy ratio $p_{v}$ as the angular fraction of the images in the field of view, the average distance from the eye to the agents $d_{v}$ estimated by the vertical angular diameter, and the minimum distance to the neighbors $d_{\min}$. As shown in Fig. 4a, $p_{v}$ is smaller and $d_{v}$ and $d_{\min}$ are larger in the order of (iii) swarm, (i) vortex, (ii) polarized school, and (iv) turning. In particular, $d_{v}$ is close to the equilibrium distances of the forces $r_{e}=2$ and $\rho_{e}=1$ for (i) and (ii), (iv), respectively.

To study the visual screening effect by the neighbors, we define the relative occupancy ratio $p_{n}$ of the *n*th nearest neighbor (*n*NN) as the fraction of the number of bins occupied by the image of *n*NN in the bins occupied by all neighbors. We call *n* the topological distance. As shown in Fig. 4b, $p_{n}$ decays to almost zero at n=99 for the patterns (i),(ii), and (iii), which means that an agent cannot see all the neighbors in the cluster at a time. For comparison, we also study the case where 100 virtual agents are randomly positioned in a circle with a radius which is 7 length unit. Their orientation is either random (referred to as the random-random case) or aligned (the random-aligned case). Interestingly, for both cases, $p_{n}$ reaches to a nonvanishing constant at n=99 (see Fig. 4b), even though $p_{v}$ is larger and $d_{v},d_{\min}$ are smaller than that of a swarm (see Fig. 4a). It indicates that although the school as a whole is sparse, the visual field is screened more by the neighbors that are locally gathered by attraction compared to the random case.

Next we verify the role of the visual attention in the screening effect. The probability for the angle of visual attention to lie within the bins occupied by *n*NN also reaches zero as *n* is increased (see Fig. 16d). Furthermore, the speeding force as a function of the topological distance *n* (Fig. 17) is a strong attraction for large *n*. However, because the probability for the visual attention is small for large *n*, the expected value of the force is dominated by the repulsive force from near neighbors, which occupy most of the visual field.

### Force map for pairwise and three-body interactions

**Fig. 5.**
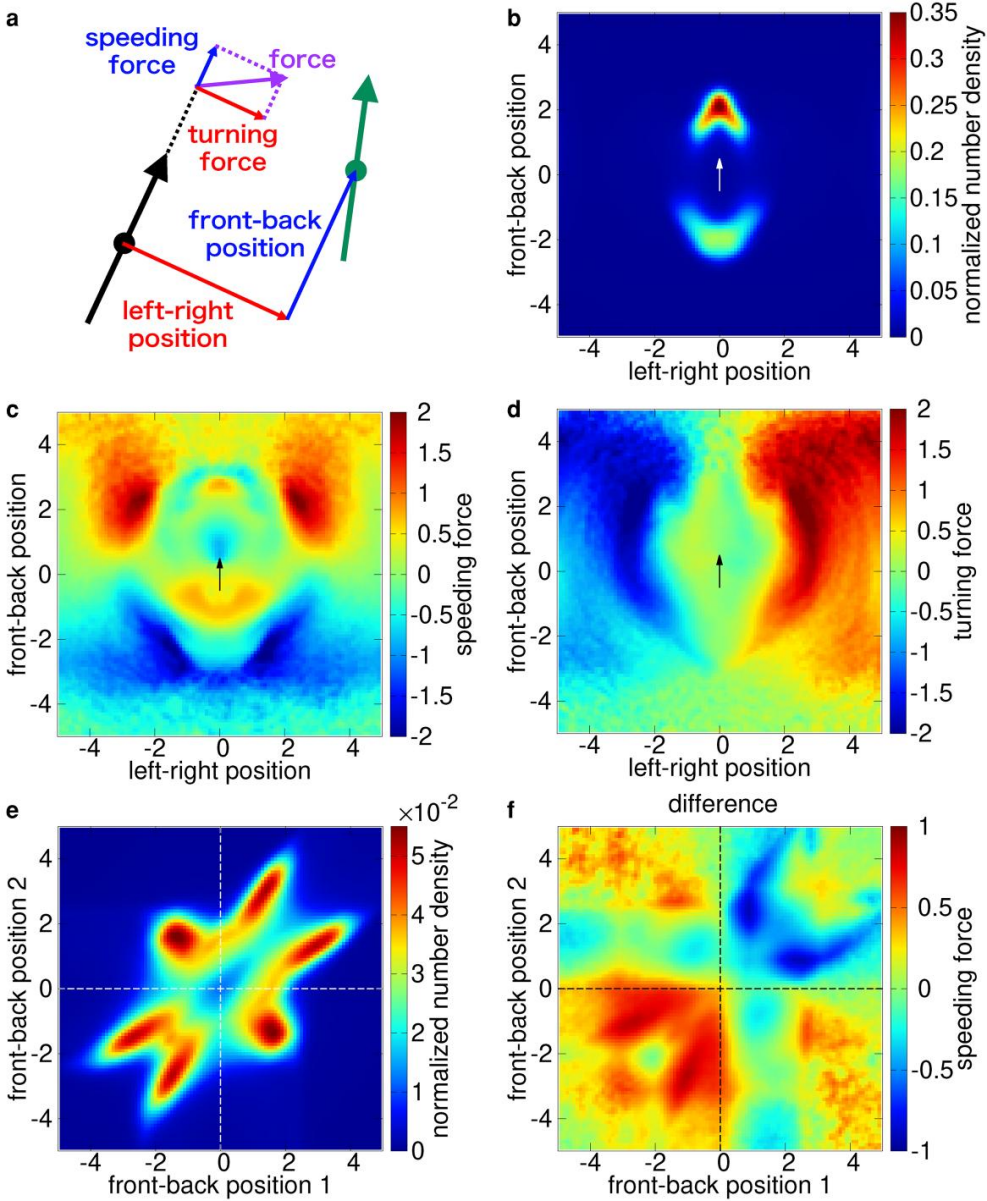
a) Definition of the force acting on the focal agent (black) and relative position of the neighbor (green). For the two-body interaction, b) the positional distribution, c) speeding force, d) turning force are mapped with respect to the relative position. The white or the black arrow in the center of each map represents the focal agent. For the three-body interaction, e) the positional distribution and f) the difference speeding force are mapped with respect to the front–back positions of neighbor 1 and neighbor 2. The parameters are $\chi =0.3,\omega_{o}=1.0$.

Finally, in the presence of one or two neighbor agents, we consider their positional distribution (number density) and forces exerted by them. They are mapped as functions of the front–back position and the left–right position of the neighbors. Following the experiment (47), we measure the force by the acceleration of the focal agent and divide it into the speeding force and turning force, which are the components parallel and perpendicular to the direction of motion, respectively (see Fig. 5a).

For two agents, the positional distribution of the neighbor has two peaks in the front and rear. The front peak is higher than the rear one, as shown in Fig. 5b. Regarding the force map, the speeding force shows strong attraction at the left- and right-front and at the center-rear (see Fig. 5c). This forward–backward asymmetry is originated from the self-propelled force. As shown in Fig. S18a, if we subtract the self-propelled force, the force map becomes forward–backward symmetric by construction of the speeding force of our model (see Eq. 11). The turning force is strong at the left- and right-front (see Fig. 5d). This is interpreted by the alignment term, which is strong at the frontal side (see Eq. 16). Moreover, as shown in Fig. S18b,d, the attractive speeding force and the attractive turning force increase with the relative speed of the neighbor, although we did not explicitly introduce such a dependence in the model Eqs. 5, 6. (See supplementary material Text and Figs. S18c,e for the relative heading dependence of a neighbor.)

Next, we focus on the three-body interaction. In particular, we analyze the relation between the front–back positions of the two neighbors and the three-body interaction. As shown in Fig. 5e, the normalized number density exhibits a characteristic aster pattern: this pattern indicates that the three agents tend to make a line-formation with a certain front–back distance between each pair. The speeding force of the three-body interaction nearly vanishes at the equilibrium position which corresponds to the peak of the normalized number density (see Fig. S19d). To clarify the nature of the three-body interaction, we subtract the averaged pairwise speeding forces from the total force. The pairwise forces are calculated assuming that only one neighbor exists at a time (see Fig. S19e). As shown in Fig. 5f, the difference force reinforces the repulsion when both neighbors are at the front or rear of an agent, and also generates a restitution force when the two neighbors are in the front and rear. Furthermore, the turning component of the difference force is relatively small compared to the speeding component (see Fig. S19i).

## Discussion

Our agent-based model incorporates selective decision-making and collective motion. The visual signal is inspired by the behaviors of ganglion cells and visual system. In particular, we introduced the motion of the direction of visual attention. The interaction forces are modeled according to the experimental knowledge, and are averaged over the resolution region of the visual attention, instead of the conventional pairwise interactions with all neighbors.

To confirm that our model actually shows the selective decision-making, we used the virtual agents method. A focal agent selects a virtual agent spontaneously with the dynamical back-and-forth motion (see Fig. S3a,d), and the marginal distributions exhibit the bifurcation behavior (see Fig. 2c–d) similar to those observed in fish and insects (19). In the case of zebrafish, the two- and three-way bifurcation for two and three virtual fish, respectively, occurs at L∼6 BL (19). On the other hand, the bifurcation occurs at L∼3 BL in our model. The difference might be explained by the resolution of the eyes; fish with a bigger body (e.g. golden shiner) has better resolution than zebrafish (48), for which our model might show a better agreement. In the presence of three virtual agents, the probability near the center one becomes small for large *L*, in agreement with the zebrafish experiment (45). For an asymmetrical configuration of virtual agents, the probability distribution shows three peaks (see Fig. S8g), which also catches the tendency for zebrafish (19). Furthermore, we have shown clearly that the conventional model which simply averages pairwise interactions from neighbor fish cannot reproduce the bifurcation process (see Fig. 2e–f).

Our model shows various patterns of collective motion (vortex, polarized school, swarm, and turning) as shown in Fig. 3a. The size of the vortex and polarized school with 100 agents is about ∼10 BL, in agreement with the size of collective patterns of 70 and 150 golden shiners (read from the Figures and Movies in Ref. (4)). In the turning pattern, the cluster rapidly turns (see Fig. S9a), and it is different from the turning phase found in the previous hydrodynamic model (7) that maintains the curved shape: the long-range hydrodynamic interaction acts on each agent as a mean field from all neighbors, and therefore a turning can occur only collectively. In contrast, for the visual interaction, the motion is affected by a small number of neighbors as in the topological interaction. When the leading agent in a cluster changes its direction, followers sensitively change the direction. Therefore, the collective motion is more sensitive to noise and it is difficult to maintain the turning pattern. The parameter *χ* that characterizes the anisotropy of the ganglion cell density is estimated as about 1/3 from an experiment (see Table S2 and supplementary material Text), and the vortex, polarized school, and swarm emerge in this *χ* region (see Fig. 3b). Regarding the dependence on the number of agents *N*, the cluster is stable for 50≲N≲200, while a larger cluster tends to split into small clusters (see Fig. S15). The instability might be caused by confinement in 2D (7, 33); in fact, a 3D model can reproduce a stable vortex for up to 10,000 agents (30, 31). For N≲50, we obtained frequent splitting, but it might be suppressed by the noise-governed interaction method as hypothesized for cichlid (*Etroplus suratensis*) (59).

The ratio of the visual field which is not filled by the other agents is $1-p_{v}∼0.2$ (see Fig. 4a), which is consistent with the ratio ∼0.2–0.4 for 70–151 golden shiners (60). In a cluster, the visual field of an agent is screened more by the neighbors comparing with the random configuration (see Fig. 4b). Regarding the topological distance, the first nearest neighbor contributes most to the repulsive speeding force and screening of the attraction (see Fig. S17c). On the other hand, for the angular velocity is not determined solely by the nearest neighbor, but several neighbors contribute nearly equally. (see Fig. S17d). In fact, the first nearest neighbor dominates the interaction in the case of mosquitofish (17), and this dominance might be also responsible for the strong repulsive speeding force compared with the attractive speeding force and the turning force in a school of golden shiner (47). Some previous models assumed the topological interaction with a fixed number of neighbors (28–31) or using Voronoi tessellation (7, 32–36). For example, a three-dimensional model with only nearest-neighbor interactions can reproduce various collective patterns (ball, torus, and school) (30). Our present model has explained the topological nature of the interaction by visual screening.

Next we compare the positional distribution of neighbor fish and the force map, with the experimental results on golden shiner (47). For the positional distribution, our results reproduce the two peaks in the front and rear including their distances ∼2 BL. We also reproduced the aster pattern in the correlation map of the front–back position of two neighbors (see Fig. 5e). In our model, the neighbor density is high in the front for both two and three agents (see Figs. 5b, S19a), but golden shiner shows a forward–backward symmetrical distribution. Also, the correlation map of the left–right positions of two neighbors shows a peak at the center in our model (Fig. S19f), but the peak is split in the experiment.

We plotted the force map by the same method used in the experiment. For the speeding force, our model reproduces the small peaks in the front and rear due to the repulsion and broad peaks in the far region due to the attractive force. However, the experimental force map has a front–back symmetry of the speeding force, while our model shows asymmetrical patterns. The symmetry might be reproduced by incorporating anisotropic interactions in the present model. For dependence on the relative speed of a neighbor, the attraction increases with the relative speed (see Fig. S18b,d), which is consistent with the experimental results. For the three-body interaction, we reproduced the restitution speeding force (see Fig. 5f) and the fact that the turning force is given by the averaged pairwise force (see Fig. S19i). However, the reinforcement of the repulsive speeding force by the front or rear neighbors (see Fig. 5f) is small or vanishing in the experiment.

In summary, our visual model provides a step beyond phenomenological models, and comprehensively reproduces the cooperative behaviors of fish, and gives insight on decision-making, collective motion, topological nature of the interaction, and positional and force distributions. Our results are mostly consistent with the experiments, but additional elements in the interaction would be necessary to resolve the remaining issues. Inclusion of (i) complex neural system for integrating information from the left and right eyes (51), (ii) more precise dependences on the angular position and relative heading of a neighbor (57), (iii) hydrodynamic signals via the lateral line (6), and (iv) direct hydrodynamic interactions by the reverse Kármán vortex (8, 9) will be interesting future directions.

## Materials and methods

Here, we show the details of our model and the order parameters. See supplementary material Text for more details of the definition of formulas, parameters, quantities measured in the simulation, measuring method, and the other information.

### The visual signal

The ganglion cell density is represented by *D*, which is a periodic function of $\phi_{\mu }$:


(7) $$D(\phi_{\mu };\chi )=\frac{1+\chi \cos\;\phi_{\mu }}{1+\chi },$$

where χ∈[0,1] is the anisotropy parameter. The ratio between the cell densities at the front and rear side of the retina is given by D(±π;χ)=(1−χ)/(1+χ).

Dependence on the vertical angular diameter is given by the function


(8) $$A(\delta_{i,\mu }^{⊥})=\hat{A}\frac{r_{0}}{r_{0}+r_{i,\mu }},$$

where


(9) $$r_{i,\mu }=\frac{h_{b}}{2\tan\;(\delta_{i,\mu }^{⊥}/2)}$$

is the relative distance of a neighbor, and $r_{0}$ is the limiting distance at which a body length can be identified. Thus the amplitude of a signal decreases as the relative distance increases.

Dependence on the relative speed of a neighbor is described by


(10) $$U(\delta_{i,\mu }^{⊥},u_{i,\mu };\beta )=(1-e^{-r_{i,\mu }/r_{a}})+e^{-r_{i,\mu }/r_{a}}e^{\beta \{(u_{i,\mu }/v_{0})-1\}}$$

is an increasing function of $u_{i,\mu }$ controlled by β≥0. Here, $r_{a}$ corresponds to the distance at which the pattern on the body of a neighbor can be detected and the speed can be measured. When the neighbor is in the far distance (compared with $r_{a}$), the amplitude of a signal is almost independent of speed (U≃1) because it is difficult to perceive the neighbor’s speed. On the other hand, when the neighbor is close enough, the amplitude increases as $U≃e^{\beta \{(u_{i,\mu }/v_{0})-1\}}$ for a neighbor with a large relative speed, and tends to increase for larger *β*.

### The equation of motion

The formula for the force and angular velocity is based on Refs. (17, 47, 57). The speeding force in Eq. 5 is given by


(11) $$F(\phi_{\mu },\delta_{i,\mu }^{⊥})=f(r_{i,\mu })\cos\;(\phi_{\mu }),$$

(12) $$\begin{array}{rl} \;f(r) & =\left\{ \begin{array}{cc} -f_{r}\frac{r_{e}-r}{r_{e}} & [0<r<r_{e}],\\ f_{a}\frac{r-r_{e}}{r_{a}-r_{e}} & [r_{e}<r<r_{a}],\\ f_{a}\frac{r_{a}}{r} & [r_{a}<r], \end{array}\right. \end{array}$$

where $r_{e}$ is the equilibrium distance and $r_{a}$ is the distance that gives the maximum attractive force $f_{a}$. When $r_{i,\mu }=0$, *f* gives the maximum repulsive force $-f_{r}$.

The angular velocity *Ω* in Eq. 6 consists of the repulsion–attraction term $\Omega_{r,a}$ and the alignment term $\Omega_{o}$, as


(13) $$\Omega (\phi_{\mu },\delta_{i,\mu }^{⊥},\psi_{i,\mu })=\Omega_{r,a}(\phi_{\mu },\delta_{i,\mu }^{⊥},\psi_{i,\mu })+\Omega_{o}(\phi_{\mu },\delta_{i,\mu }^{⊥},\psi_{i,\mu }).$$

The repulsion–attraction term is


(14) $$\Omega_{r,a}(\phi_{\mu },\delta_{i,\mu }^{⊥},\psi_{i,\mu })=\omega (r_{i,\mu })\sin\;\phi_{\mu }\left(1-e^{-r_{i,\mu }/r_{a}}\frac{1+\cos\;\psi_{i,\mu }}{2}\right),$$

(15) $$\begin{array}{rl} & \omega (r)=\left\{ \begin{array}{cc} -\omega_{r}\frac{\rho_{e}-r}{r_{e}} & [0<r<\rho_{e}],\\ \omega_{a}\frac{r-\rho_{e}}{r_{a}-\rho_{e}} & [\rho_{e}<r<r_{a}],\\ \omega_{a}\frac{r_{a}}{r} & [r_{a}<r], \end{array}\right. \end{array}$$

where ω(r) is similar to f(r), and $\rho_{e}$ is the equilibrium left–right distance, which is smaller than $r_{e}$ due to the slender body of fish. We assume that fish can precisely detect the relative heading as well as the relative speed of a neighbor within $r_{a}$. The relative heading dependence behaves as $(1-\cos\;\psi_{i,\mu })/2$ at near region, and reaches 1 at far region. The alignment term is


(16) $$\Omega_{o}(\phi_{\mu },\delta_{i,\mu }^{⊥},\psi_{i,\mu })=\omega_{o}\text{exp}\left\{-\frac{(r_{i,\mu }-r_{o}{)}^{2}}{2l_{o}^{2}}\right\}\frac{1+\cos\;\phi_{\mu }}{2}\sin\;\psi_{i,\mu },$$

where $\rho_{e}<r_{o}<r_{a}$, $l_{o}$ is the characteristic length, and $\omega_{o}$ is the strength of alignment.

Finally, $\left\langle Q(q_{i,\mu })\rangle_{\mu }=\sum_{\mu \in R_{i}}Q(q_{i,\mu })/|R_{i}\right|$ is the average of a quantity $Q(q_{i,\mu })$ in the resolution angle region centered on $\phi_{i}$: a set of bins $R_{i}$ includes the bin in $\left[\phi_{i}-\delta_{0}^{∥}/2,\phi_{i}+\delta_{0}^{∥}/2\right]$, and $\left|R_{i}\right|$ is its size. The horizontal resolution angular diameter $\delta_{0}^{∥}$ is related to $r_{0}$ as $\delta_{0}^{∥}=2\tan^{-1}\;[l_{b}/(2r_{0})]$. In other words, the horizontal resolution angular diameter corresponds to the angular diameter created by a body length at the limiting distance $r_{0}$.

### The order parameters

The polar order parameter is defined as


(17) $$P(t)=\frac{1}{N}\left|\sum_{i=1}^{N}e_{i}(t)\right|.$$

It takes the maximal value 1 when the agents are completely aligned, and vanishes when the orientation of the agents is completely random. The rotational order parameter is defined as


(18) $$M(t)=\frac{1}{N}\left|\sum_{i=1}^{N}{\hat{c}}_{i}(t)\times e_{i}(t)\right|,$$

where ${\hat{c}}_{i}(t)=(r_{i}(t)-r_{G}(t))/|r_{i}(t)-r_{G}(t)|$ is the unit vector that gives the direction from the center of mass position $r_{G}=\sum_{i=1}^{N}r_{i}/N$. The rotational order parameter is large when the agents are rotating around a common axis in the same direction.

## Supplementary Material

pgae264_Supplementary_Data

## Data Availability

All data that support the findings of this study are available in the manuscript and the Supplementary material.

## Preprints

This manuscript was posted on a preprint: https://doi.org/10.48550/arXiv.2402.09073.
