# Supplementary material for: Selective decision-making and collective behavior of fish by the motion of visual attention
Source: PNAS Nexus. 2024 Jul 2;3(7):pgae264. doi: 10.1093/pnasnexus/pgae264 (PMC11264410; doi:10.1093/pnasnexus/pgae264)
Supplement: pgae264_Supplementary_Data [file pgae264_supplementary_data.zip › PNASNEXUS-PNASNEXUS-2024-00298-TR-s01.pdf]

Supplementary Information for "Selective decision making and collective behavior of fish by the motion of visual attention"

Susumu Ito, and Nariya Uchida*
Department of Physics, Tohoku University, Sendai, 980-8578, Japan
(Dated: June 28, 2024)

CONTENTS

I. Model 2
  A. Comparison with previous visual models 2
  B. Dynamical equations 3
    1. The visual field 3
    2. The visual signal 5
    3. Forces 6
  C. Parameters 8
  D. Numerical integration of equation 11

II. Behavior of selective decision making 12
  A. Definition of probability distribution 12
  B. Parameter dependence and asymmetrical configuration 12
  C. Conventional particle model 13

III. Collective motion 18
  A. Definition of the splitting 18
  B. Cluster size and duration of the order 18
  C. Dependence on the initial condition 19
  D. Parameter dependence of the collective pattern 19

IV. Visual information and topological distance 26
  A. Occupancy ratio and distance to neighbors 26
  B. Functions of the topological distance 26

V. Force map for pairwise and three-body interactions 29
  A. Definitions of the positional distribution and forces 29
  B. Maps for two agents 29
  C. Maps for three agents 29

VI. Supplemental Movies 32

References 32

Note: The titles of Sect.I-V correspond to those in the main text. The Supplementary Figures, Tables, and Equations are contained in each section.

---

* uchida@cmpt.phys.tohoku.ac.jp

I. MODEL

A. Comparison with previous visual models

Previous models of visual interactions are summarized in Table S1. First, there are several agent-based models that integrate the visual information from all detected neighbors.

- Ref. [1] uses a 2D model for fish schools incorporating occlusion by the horizontal image of a neighbor. The agent is line-shaped. The visual field is divided into bins, and the agent interacts with the closest neighbor in each bin. Each agent determines its speed by Gaussian white noise, and the angular velocity by the average of the pairwise interaction with the neighbors.
- Ref. [2] proposed a 2D/3D model for bird flocks with circle/sphere-shaped agents. The model uses the angles of the edges of the images of neighbors for attraction, in addition to the topological interaction for alignment, and considers occlusion. Each agent has constant speed.
- Ref. [3] introduced the threshold of strength of visual information (without occlusion) in a 2D model. The speed is constant, and the angular velocity is determined by averaging the contribution of the neighbors whose angular diameter and horizontal speed exceed the thresholds. Topological interactions are added to the model in Ref. [4].
- Ref. [5] considers a 2D/3D model in which both the speed and the angle change by interactions, and incorporates occlusion. The shape of an agent is a circle/sphere. The model uses the horizontal size and position of the edges the images, and the visual information is integrated. The speeding force and the turning force incorporate the symmetry of the interaction of fish. Ref. [6] uses a 2D model with the modified distance function.
- Ref. [7] proposed a 2D model that incorporates the temporal change of the size of images ("optical flow"). the optical flow which expresses the temporal change of the size of image. The agent has a circular shape and constant speed. Occlusion is considered, and the angular velocity is determined by integrating the visual information.

Decision making is treated in two previous models:

- Ref. [8] considers a quasi-2D model, where each agent moving on a plane consists of three line segments representing the body length, height, and width of zebrafish. By using the height of the agent, not only its horizontal size, the model prevents overestimation and/or underestimation of the number of neighbors. The moving direction of the agent is determined by a probability distribution function at each time step. The time step corresponds to the period of burst-coast swimming of zebrafish. The distribution is given by superposition of a von Mises distribution for each neighbor, whose angular position and solid angle determine the peak position and height, respectively. Occlusion is not considered, while attractive interactions are included. The agents are in heterogeneous environments consisting of a square tank and feeders.
- Ref. [9] introduced a 2D model of point-like agents, with a focal agent following two or three virtual agents. The focal agent has a network of spins inspired by the 1D Ising model: the relative position of a virtual agent determines a goal vector that corresponds to a spin, and the interaction between spins is determined by the angle between the goal vectors. A phase transition occurs when the distance between the virtual agents becomes sufficiently large. When they are equally distanced from the focal agent, one of them is selected by spontaneous symmetry breaking. Ref. [10] presented a detailed theoretical analysis of the model, and Ref. [11] simplified the model by coarse-graining the spin dynamics.

To the best of our knowledge, our model is the first to treat decision-making and collective motion simultaneously. By introducing the direction of visual attention, we could describe the limited and dynamically evolving visual

information for each agent. Various patterns of collective motion are reproduced by considering repulsion, attraction, and orientational interaction between agents. The details of our model is shown in the main text and below.

TABLE S1. List of the visual models related to the present work.

reference	dimension	number of agents	shape of agent, treatment of occlusion	information of neighbors	variables of equation of motion	interaction type	force type
Kunz and Hemelrijk (2012) [1]	2D	10-10000	line, Yes	position	angle	average of pairwise interactions	repulsion, attraction, reorientation
Pearce *et al.* (2014) [2]	2D/3D	100-1000	circle/ball, Yes	edge of image, topological distance	angle	average of edge angle, average of direction of motion	attraction to edge, reorientation
Lemasson *et al.* (2009) [3]	2D	25	a certain size body, No	size of image, speed of image	angle	summation of pairwise interactions	repulsion, attraction, reorientation
Bastien and Romanczuk (2020) [5]	2D/3D	2-100	circle/ball, Yes	size and edge of image	speed, angle	integral of force with respect to visual field	repulsion, attraction
Castro *et al.* (2023) [7]	2D	10-300	circle, Yes	size of image, optical flow	angle	integral of force with respect to visual field	repulsion, attraction, reorientation
Collignon *et al.* (2016) [8]	quasi-2D	10	three line segments, No	solid angle of image	angle	stochastic process	attraction
Sridhar *et al.* (2021) [9]	2D	1 (and 2-3 virtual agents)	point particle, No	angle between neighbors	angle	phase transition dynamics	attraction
Present work	quasi-2D	2-500	plate, Yes	size of image, speed of image, relative heading angle	speed, angle	angle of visual attention	repulsion, attraction, reorientation

B. Dynamical equations

1. *The visual field*

The self-propelled agent in our model is a plate with a body length l_b and a body height h_b. As shown in Fig. 1(a), the eye is located at distance l_e from the center of body toward the head, and its position is denoted as $\boldsymbol{r}_i$. The visual field is divided into N_b bins indexed by $\mu = 1, 2, \cdots, N_b$. The μ-th bin is centered at the angle ϕ_μ and has the angular width $\delta_b^{\parallel} = 2\pi/N_b$. (see Fig. 1(d)). The vertical angular diameter $\delta_{i,\mu}^{\perp}$, the relative speed $u_{i,\mu}$, and the relative heading angle $\psi_{i,\mu}$ of the neighbor detected at the μ-th bin of the agent i are formulated as follows.

Given the focal agent i and its neighbor j, we calculate the vertical angular diameter from the eye of i to a part of

j's body at the angle ϕ as

$$\delta_{ij}^{\perp}(\phi) = 2\tan^{-1}\left(\frac{h_b}{2r_{ij}(\phi)}\right), \tag{S1}$$

where $r_{ij}(\phi)$ is the distance between i's eye and j's body as shown in Fig. S1(a),(d). In order to obtain $r_{ij}(\phi)$, we use the relative position vector

$$\boldsymbol{r}_{ij}(\phi) = c_1\boldsymbol{\varepsilon}_i(\phi) = \boldsymbol{r}_j - \boldsymbol{r}_i + c_2\boldsymbol{e}_j, \tag{S2}$$

where $\boldsymbol{\varepsilon}_i(\phi) = (\cos(\theta_i + \phi), \sin(\theta_i + \phi))$, $\boldsymbol{e}_j = (\cos\theta_j, \sin\theta_j)$, and c_1 and c_2 are calculated as

$$\begin{bmatrix} c_1 \\ c_2 \end{bmatrix} = \frac{1}{\sin(\theta_i - \theta_j + \phi)} \begin{bmatrix} -\sin\theta_j & \cos\theta_j \\ -\sin(\theta_i + \phi) & \cos(\theta_i + \phi) \end{bmatrix} \begin{bmatrix} x_j - x_i \\ y_j - y_i \end{bmatrix}. \tag{S3}$$

(see Fig. S1(b)). Substituting the expression of c_1 to Eq. (S2), we obtain

$$r_{ij}(\phi) = |\boldsymbol{r}_{ij}(\phi)| = |c_1| = \left|\frac{(-\sin\theta_j, \cos\theta_j)\cdot(\boldsymbol{r}_j - \boldsymbol{r}_i)}{\sin(\theta_i - \theta_j + \phi)}\right|. \tag{S4}$$

Next, we maximize the vertical angle $\delta_{ij}^{\perp}(\phi)$ in the μ-th bin ($\phi \in [\phi_\mu - \delta_b^{\parallel}/2, \phi_\mu + \delta_b^{\parallel}/2]$), and denote it as $\delta_{ij,\mu}^{\perp}$. We define the unit vectors $\boldsymbol{e}_{ij}^{\mathrm{an}}$, $\boldsymbol{e}_{ij}^{\mathrm{pos}}$ pointing to the edge of the anterior and posterior of j, respectively (see Fig. S1(c)):

$$\boldsymbol{e}_{ij}^{\mathrm{an}} = \frac{\boldsymbol{r}_j - \boldsymbol{r}_i + \left(\frac{l_b}{2} - l_e\right)\boldsymbol{e}_j}{\left|\boldsymbol{r}_j - \boldsymbol{r}_i + \left(\frac{l_b}{2} - l_e\right)\boldsymbol{e}_j\right|},\ \boldsymbol{e}_{ij}^{\mathrm{pos}} = \frac{\boldsymbol{r}_j - \boldsymbol{r}_i - \left(\frac{l_b}{2} + l_e\right)\boldsymbol{e}_j}{\left|\boldsymbol{r}_j - \boldsymbol{r}_i - \left(\frac{l_b}{2} + l_e\right)\boldsymbol{e}_j\right|}. \tag{S5}$$

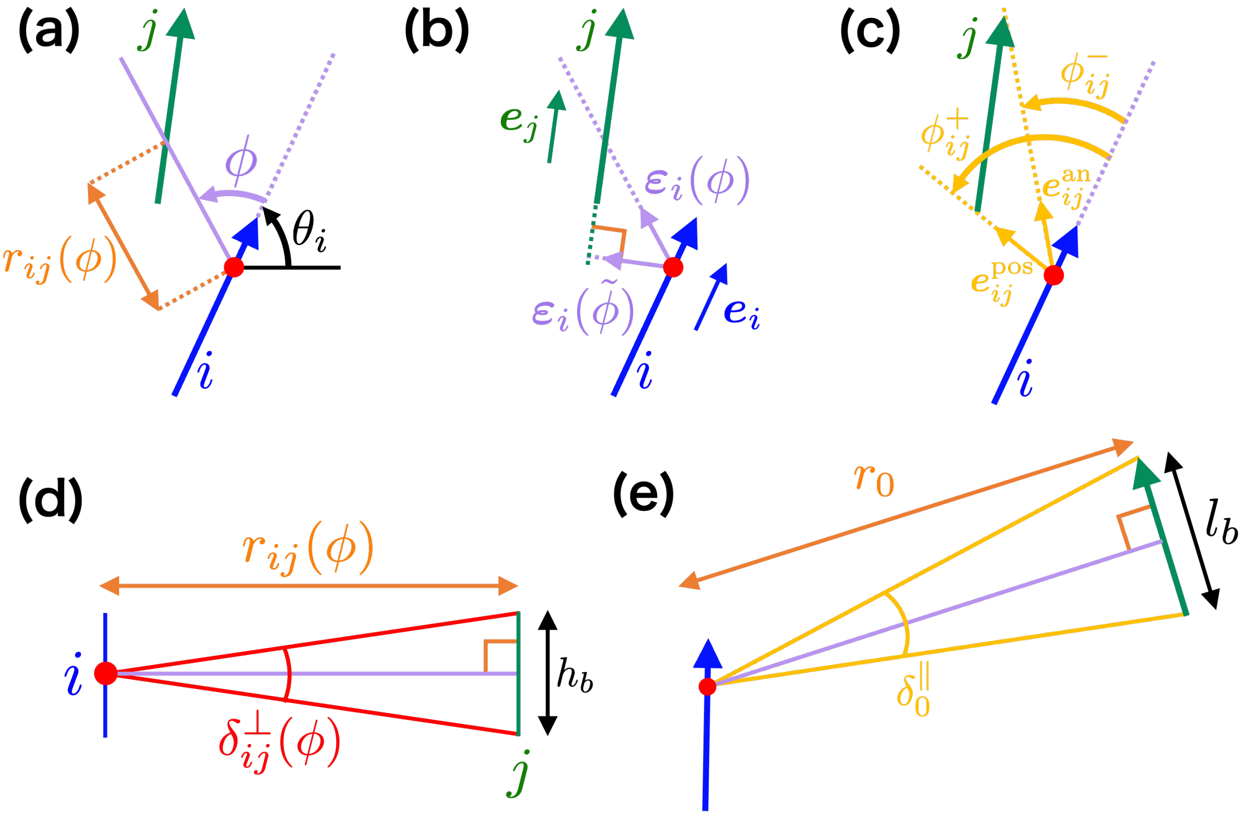

FIG. S1. The quantities related to the visual field in our model. The red point is the eye of the agent i. (a)-(c) Relation between the distance, vectors, and angles. (d)-(e) Relation between the distance and angular diameter. (d) shows the vertical cross section containing the purple solid line at the angle ϕ in (a).

We relabel $\boldsymbol{e}_{ij}^{\mathrm{an}}$ and $\boldsymbol{e}_{ij}^{\mathrm{pos}}$ as $\boldsymbol{e}_{ij}^{+}$ and $\boldsymbol{e}_{ij}^{-}$ so that $(\boldsymbol{e}_{ij}^{-} \times \boldsymbol{e}_{ij}^{+}) \cdot \boldsymbol{e}_z > 0$ is satisfied ($\boldsymbol{e}_z$ is the unit vector along the z-axis). For example, in the case of Fig. S1(c), $\boldsymbol{e}_{ij}^{\mathrm{an}} = \boldsymbol{e}_{ij}^{-}$ and $\boldsymbol{e}_{ij}^{\mathrm{pos}} = \boldsymbol{e}_{ij}^{+}$. The angles between $\boldsymbol{e}_{ij}^{\pm}$ and $\boldsymbol{e}_i$ are given by

$$\phi_{ij}^{\pm} = \mathrm{sgn}\left((\boldsymbol{e}_i \times \boldsymbol{e}_{ij}^{\pm}) \cdot \boldsymbol{e}_z\right) \cos^{-1}\left(\boldsymbol{e}_i \cdot \boldsymbol{e}_{ij}^{\pm}\right), \tag{S6}$$

where $\mathrm{sgn}\,(\circ)$ is the sign function which takes ± 1, and $\phi_{ij}^{\pm} \in [-\pi, \pi]$. To obtain $\delta_{ij,\mu}^{\perp}$, we need to calculate the unit vector $\boldsymbol{\varepsilon}_i(\tilde{\phi})$, the line along which is perpendicular to the line extending the body of j, as shown in Fig. S1(b). Considering the direction of j, we obtain it as

$$\boldsymbol{\varepsilon}_i(\tilde{\phi}) = \mathrm{sgn}\left((\boldsymbol{e}_{ij}^{\mathrm{an}} \times \boldsymbol{e}_{ij}^{\mathrm{pos}}) \cdot \boldsymbol{e}_z\right) R_{\frac{\pi}{2}} \boldsymbol{e}_j,\ R_{\frac{\pi}{2}} = \begin{bmatrix} 0 & -1 \\ 1 & 0 \end{bmatrix}. \tag{S7}$$

The maximal vertical diameter $\delta_{ij,\mu}^{\perp}$ depends on the sign of the cross products $s_{ij}^{\pm} = (\boldsymbol{\varepsilon}_i(\tilde{\phi}) \times \boldsymbol{e}_{ij}^{\pm}) \cdot \boldsymbol{e}_z$ as follows.

- case 1: $s_{ij}^{+} > 0$ and $s_{ij}^{-} < 0$. In this case, the extended line of $\boldsymbol{\varepsilon}_i(\tilde{\phi})$ intersects j's body.
  - case 1-1: If the μ-th bin contains $\tilde{\phi}$ (in other words, $\tilde{\phi} \in [\phi_\mu - \delta_b^{\parallel}/2, \phi_\mu + \delta_b^{\parallel}/2]$), then $\delta_{ij,\mu}^{\perp} = \delta_{ij}^{\perp}(\tilde{\phi})$.
  - case 1-2: If not, then $\delta_{ij,\mu}^{\perp} = \delta_{ij}^{\perp}(\phi')$, where ϕ' is the angle that is closest to $\tilde{\phi}$ in the μ-th bin.
- case 2: $s_{ij}^{+} > 0$ and $s_{ij}^{-} > 0$. In this case, the extended line of $\boldsymbol{\varepsilon}_i(\tilde{\phi})$ intersects the extended line of j's body at the minus side of j.
  - case 2-1: If the bin μ contains ϕ_{ij}^{-}, then $\delta_{ij,\mu}^{\perp} = \delta_{ij}^{\perp}(\phi_{ij}^{-})$.
  - case 2-2: If not, then $\delta_{ij,\mu}^{\perp} = \delta_{ij}^{\perp}(\phi')$.
- case 3: $s_{ij}^{+} < 0$ and $s_{ij}^{-} < 0$. In this case, the extended line of $\boldsymbol{\varepsilon}_i(\tilde{\phi})$ intersects the extended line of j's body at the plus side of j. This case is illustrated in Fig. S1(b),(c).
  - case 3-1: If the bin μ contains ϕ_{ij}^{+}, then $\delta_{ij,\mu}^{\perp} = \delta_{ij}^{\perp}(\phi_{ij}^{+})$.
  - case 3-2: If not, then $\delta_{ij,\mu}^{\perp} = \delta_{ij}^{\perp}(\phi')$.

Finally, we obtain $\delta_{i,\mu}^{\perp}$ as the maximum vertical angular diameter among all neighbors in the μ-th bin:

$$\delta_{i,\mu}^{\perp} = \max_{j=1,\cdots,N} \delta_{ij,\mu}^{\perp}. \tag{S8}$$

Let $J_{i,\mu}$ be the label of the neighbor that gives the maximum vertical angular diameter $\delta_{i,\mu}^{\perp}$ at the μ-th bin for the agent i. The relative speed of the neighbor in the μ-th bin is defined by

$$u_{i,\mu} = \left|\boldsymbol{v}_{J_{i,\mu}} - \boldsymbol{v}_i\right|. \tag{S9}$$

Similarly, the relative heading angle $\psi_{i,\mu}$ of the neighbor is

$$\psi_{i,\mu} = \mathrm{sgn}\left((\boldsymbol{e}_i \times \boldsymbol{e}_{J_{i,\mu}}) \cdot \boldsymbol{e}_z\right) \cos^{-1}\left(\boldsymbol{e}_i \cdot \boldsymbol{e}_{J_{i,\mu}}\right). \tag{S10}$$

Note that the above calculations do not consider the possible intersection of a focal agent and a neighbor.

2. The visual signal

We use the height of the agent for formulation of the visual signal, because it is shown experimentally that zebrafish mainly use the vertical size of neighbors' images [12], and also because use of the horizontal size only leads to overestimation and/or underestimation of the number of neighbors [8]. The relative speed also plays an important role as mentioned in the main text [13, 14]. Therefore, we use the vertical angular diameter and the relative speed to determine the signal. The term "signal" in this paper corresponds to the stimulus in the context of biology. More

precisely, a signal corresponds to the electrical signal that encodes the visual information such as the size and velocity of the image detected by the ganglion cells [13]. In the context of physics, the signal corresponds to the potential that induces the motion of the angle of visual attention. The angle of visual attention $\phi_i \in [-\pi, \pi]$ moves toward a potential minimum created by a strong signal that reflects large size and/or relative speed of the images.

The signal consists of the four functions G, D, A, and U (see Eq. (1) and Eq. (3) and the main text). The functions G and D characterize the eyeball system and do not depend on the visual information ($\delta^{\perp}_{i,\mu}$, $u_{i,\mu}$). On the other hand, A and U correspond to the reaction to the visual information ($\delta^{\perp}_{i,\mu}$, $u_{i,\mu}$). Each function is formulated as follows.

- The function G gives the angular distribution of the visual signal around the bin where the images are detected. It is a von Mises distribution for the angular distance $|\phi - \phi_\mu|$ with the sharpness parameter κ, and is normalized so that $G = 1$ at $\phi = \phi_\mu$ (see Eq. (2)).

- The function D represents the distribution of the density of ganglion cells, which is higher on the rear side of the retina [15]. It means that the resolution of the eye is higher when looking forward than backward (which is consistent with the observation that fish tend to follow neighbors in the front [14]). Therefore, D has the normalized maximum value $D = 1$ in the front ($\phi_\mu = 0$), decreases with ϕ_μ, and has the minimum at the rear ($\phi_\mu = \pm\pi$) (see Eq. (7)). The degree of anisotropy is given by the parameter χ, and the ratio of ganglion cell densities at the front and rear is $D(\pm\pi; \chi)/D(1; \chi) = (1 - \chi)/(1 + \chi)$.

- The function A gives the amplitude that depends on the vertical angular diameter (see Eq. (8)). It has the reference amplitude $\hat{A}$ at $r_{i,\mu} = 0$ (or $\delta^{\perp}_{i,\mu} = \pi$), and then decreases as the size of the image decreases. Therefore, the angle of visual attention ϕ_i is attracted toward a closer neighbor, in consistent with the observation that fish tend to follow a closer neighbor [14]. The characteristic decay length of the amplitude is set equal to the limiting distance at which a body length can be identified [15], $r_0 = l_b / \left[2 \tan\left(\delta^{\parallel}_0/2\right)\right]$. Here, $\delta^{\parallel}_0$ is the minimal horizontal angular diameter of the image that fish can detect, which corresponds to a body length l_b at the distance r_0; see Fig. S1(e). Therefore, it would be reasonable to assume that the signal from a fish located beyond the limiting distance (with the horizontal angular diameter smaller than $\delta^{\parallel}_0$) is proportional to its vertical angular diameter: for $r_{i,\mu} \gg r_0$, we have $A \propto 1/r_{i,\mu} \propto \delta^{\perp}_{i,\mu}$.

- The function U describes the dependence on the relative speed of the image $u_{i,\mu}$ through the parameter β (see Eq. (10)). When $\beta = 0$, the signal has no dependence on the relative speed and we set $U = 1$. For $\beta > 0$, U is an increasing function of $u_{i,\mu}$, and the angle of visual attention ϕ_i moves toward a neighbor with a larger relative speed, which reflects the experimental result [14]. We assume $U \simeq 1$ for $r_{i,\mu} \gg r_a$, which means that a fish cannot detect the moving direction of a neighbor located beyond the characteristic distance r_a. In fact, fish can detect the pattern of the body of a neighbor inside a limiting distance [15]. Also, fish can track the vertical stripe pattern that moves horizontally [16], which implies that the fish can detect the relative speed.

An example of the synthesized signal $\Gamma_i(\phi)$ (Eq. (3)) is given in Fig. S2. The synthesized signal has some local minima, and the angle of visual attention ϕ_i is attracted by one of the minimum through the dynamical equation (4). In Fig. S2(b), the colored solid line represents the synthesized signal produced by the neighbor shown in the same color. It is the superposition of signals in the bins occupied by the neighbor,

$$\tilde{\Gamma}_i(\phi) = \sum_{\mu \in \text{a neighbor}} D(\phi_\mu; \chi) \gamma_{i,\mu}(\phi). \tag{S11}$$

3. Forces

Here we explain the dependences of the forces on the speed v_i and moving direction θ_i. The forces in Eqs. (5),(6) consist of the self-propelled term and the interaction terms. First we consider the self-propelled term $C(v_0^2 - v_i^2)$. Previous models of fish set the speed constant [17], random [1], or determined it by a force $\propto (v_0 - v_i)$ [18]. In the

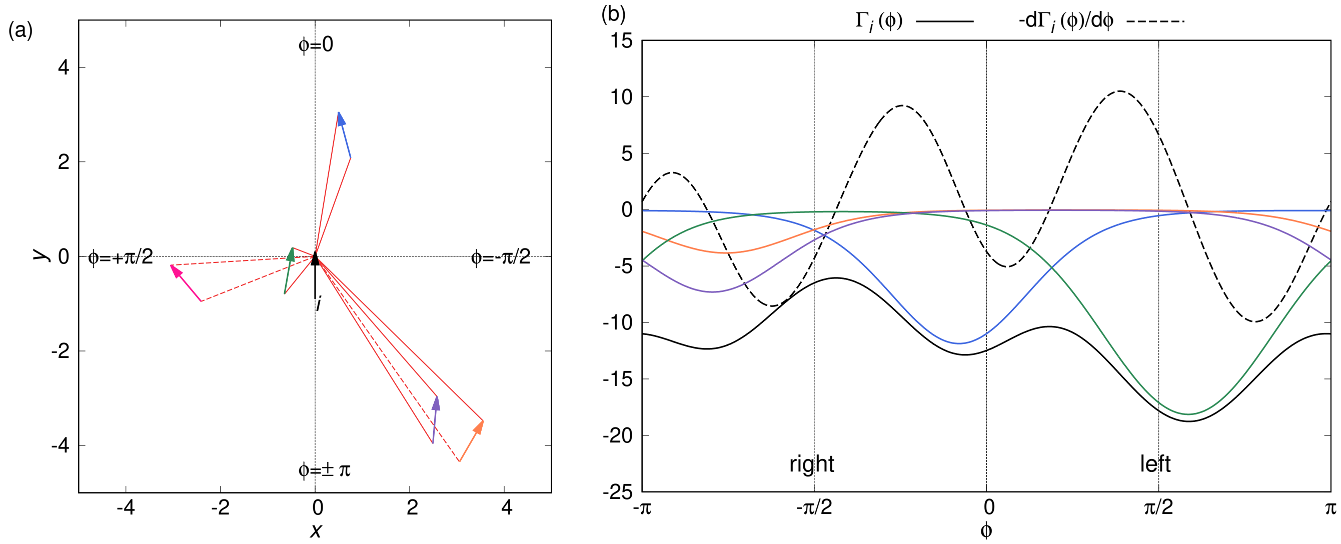

FIG. S2. An example of the synthesized signal with $\kappa = 2.5$, $\chi = 0.3$, and $\beta = 4.0$. (a) The sample configuration. For simplicity, the speed of the agents is set to v_0. The focal agent i is shown by the black arrow at the center, and the red lines connect its eye and the anterior and posterior edges of each neighbor. The dashed red lines show occlusion of images. The blue neighbor is at the right front of i. The green one is at the left side of i and completely masks the pink one. The purple one is at the right rear of i and the orange one is partially masked. (b) The synthesized signal and its derivative that correspond to the configuration of (a). The black solid line shows $\Gamma_i(\phi)$ and the black dashed line is $-\mathrm{d}\Gamma_i/\mathrm{d}\phi$. For clarity, we show the colored solid line which is the synthesized signal (Eq.(S11)) from each neighbor with the same color in (a).

present model, we take into account the fact that the steady swimming speed is determined by the balance between the drag and thrust forces [19]. For the drag force, we can assume the Newton's drag force $-Cv_i^2$ where C is constant for the Reynolds number $\sim 10^4$–10^5, and the thrust force is assumed to be constant (Cv_0^2). Note that the self-propelled force $C(v_0^2 - v_i^2)$ is approximately proportional to $v_0 - v_i$ when $v_i \simeq v_0$, reproducing the linear function in the previous model [18].

Next, we explain the interaction terms F and Ω. As mentioned in the main text, fish experiences repulsion from neighbors in near field, attraction from those in far field [20, 21] and alignment forces [22]. These forces are given as functions of the vertical angular diameter $\delta_{i,\mu}^{\perp}$ (or the corresponding distance $r_{i,\mu}$), the angle of the bin ϕ_μ, and the relative heading angle of the neighbors $\psi_{i,\mu}$ (see Eq. (S10)) as follows.

- The force F describes the repulsion and attraction, which are stronger for neighbors in the front or rear than those on the sides [20, 21]. Therefore, we factorize F into the function of distance f and the function of the relative angle $\cos\phi_\mu$ (see Eq. (11)). The function $\cos\phi_\mu$ is used also in a previous model [5], while f is based on the experimental force map [20, 21]; it gives the maximum repulsive force $-f_r$ at $r = 0$, vanishes at the equilibrium distance $r = r_e$, reaches the maximum attraction f_a at $r = r_a$, and then decreases as the distance increases (or the vertical angular diameter decreases); see Eq. (12). For $r < r_a$, we interpolate the force by a linear function of the distance, because the repulsive-attractive force increases almost linearly in near field [22]. For $r > r_a$, f is proportional to $1/r$, which also fits the experimental result well [22].

The angular velocity Ω is decomposed into the repulsion-attraction term $\Omega_{r,a}$ and the alignment term Ω_o as done in the experiment [22] (see Eq. (13)).

- The repulsion-attraction $\Omega_{r,a}$ is stronger when the neighbor is positioned on the sides [20, 21] and when the relative heading angle $\psi_{i,\mu}$ is far from zero [22]. We then factorize $\Omega_{r,a}$ into a function of the relative distance ω, the function of the relative angle of the position $\sin\phi_\mu$, and a function of the relative heading angle (see Eq. (14)). The function ω is defined in a similar manner as f, but the equilibrium distance ρ_e is shorter than r_e

reflecting the slender body of fish [20] (see Eq. (15)). The factor $\sin\phi_\mu$ is used in the previous model [5]. The dependence on the relative heading angle is described by the function

$$\Psi(r_{i,\mu},\psi_{i,\mu}) = 1 - e^{-r_{i,\mu}/r_a}\frac{1+\cos\psi_{i,\mu}}{2}, \tag{S12}$$

where we assumed that the limiting distance r_a for the agent to detect the relative heading angle is equal to the one for the relative speed; $\Psi \simeq 1$ for $r_{i,\mu} \gg r_a$. At small distances $r_{i,\mu} \sim 0$, we have $\Psi \simeq (1-\cos\psi_{i,\mu})/2$. In Ref. [22], the angular velocity is fitted by $\cos\psi_{i,\mu}$ and $\cos(2\psi_{i,\mu})$, and our formula corresponds to the first harmonic approximation.

- The alignment term Ω_o is large for a neighbor in the front and for the relative heading angle $\psi_{i,\mu} \sim \pm\pi/2$ [22]. Thus we factorize Ω_o into the function of the distance $\omega_o \exp\left\{-(r_{i,\mu}-r_o)^2/2l_o^2\right\}$, the function of the angle of the relative position $(1+\cos\phi_\mu)/2$, and the function of the relative heading angle $\sin\psi_{i,\mu}$ (see Eq. (16)). The Gaussian function for the distance gives a good approximation [22], and is peaked at a moderate distance r_o in the range $\rho_e < r_o < r_a$. The single harmonics of ϕ_μ and $\psi_{i,\mu}$ simplify the second harmonic fitting [22], similarly to that for Ψ. We do not explicitly introduce a limiting distance for detecting the relative heading angle, because the dependence on $\psi_{i,\mu}$ decreases through the Gaussian function for $r_{i,\mu} \gg r_o$.

The average $\langle\cdots\rangle_\mu$ is defined in *Materials and Methods*, and used for F and Ω in Eqs. (5),(6). It is taken over the region of angular resolution, which is centered at the angle of visual attention ϕ_i, and has the width equal to the limiting horizontal angular diameter $\delta_0^{||}$ (see Fig. S1(e)). Numerically, we define a set of bins $\mathcal{R}_i$ in the region and its size as $|\mathcal{R}_i| = 1 + 2\mu_0$, where μ_0 is an integer closest to $\delta_0^{||}/(2\delta_b^{||})$ ($\delta_b^{||} = 2\pi/N_b$ is the bin width). The biological meaning of the average is that fish cannot detect an image smaller than $\delta_0^{||}$, and therefore the force is averaged (or "blurred") over the width $\delta_0^{||}$. If the horizontal size of the image is smaller than $\delta_0^{||}$, this blurring reduces the force due to the neighbor: reaction to a very small image is diminished by the limitation of resolution.

C. Parameters

Here, we show the details of the parameters and their correspondence to the experimental values. See Table S2 for a summary of the parameters.

- An agent has the body height $h_b \sim 0.3$ BL (body length), which is the averaged value of many species of fish [23]. We set the eye position from the center of body as $l_e = 0.4$ BL; that is, the distance from the tail tip to the monoeye is 0.9 BL. The previous model sets the eye at the edge of the head for simplicity [8], but the eye position of a real fish is slightly behind.

- The visual bins are defined as follows. The density of ganglion cells is on the order of $\mathcal{O}(10^4)$ cells/mm^2 for zebrafish (*Danio rerio*) and golden shiner (*Notemigonus crysoleucas*), which use visual information as primary source of information [15]. The radius of the eye is about $\mathcal{O}(1)$ mm (see Fig. 2 in Ref. [15]), and therefore the number of ganglion cells is $\mathcal{O}(10^4)$–$\mathcal{O}(10^5)$. In our model, the number of bins in the horizontal direction is approximately the square root of the number of ganglion cells. We set $N_b = 720$ and the width of bin $\delta_b^{||} = 2\pi/N_b = 0.5°$. Therefore, each bin contains about $\sqrt{N_b}$ ganglion cells in the vertical direction, and the number of those that detected the image of a neighbor gives its vertical angular diameter.

- We define the horizontal resolution angle $\delta_0^{||} = 2°$, and this corresponds to the limiting resolution distance $r_0 = l_b/\left[2\tan\left(\delta_0^{||}/2\right)\right] \simeq 28.6$ BL. In fact, golden shiner can detect a neighbor up to the distance 16-39 BL [15]. Note that the number of bins in the resolution angle region is $|\mathcal{R}_i| = 5$ in our simulation. We set the distance for detecting the pattern of a neighbor to $r_a = 3$ BL, because zebrafish can detect the striping pattern of a neighbor at the distance 2-5 BL and golden shiner can detect the cycloid scale pattern of a neighbor at the distance 1-3 BL [15].

- Next, we explain the parameters related to the signal. The sharpness of the signal κ and the dependence of relative speed β are the control parameters. We tuned their values to reproduce the bifurcation structure in the selective decision making and various patterns of collective motion, and mostly used $\kappa = 2.5$ and $\beta = 4.0$. Regarding χ, $(1-\chi)/(1+\chi)$ represents the ratio of the density of ganglion cell between front and back as mentioned above. This ratio is about 1/2 for zebrafish and golden shiner [15], and then we can estimate $\chi \sim 1/3$, but we set χ as the control parameter. We assumed that the timescale τ_ϕ for the motion of the angle of visual attention ϕ_i is close to the timescale of steady swimming τ_0, and used $\tau_\phi = \tau_0 = 1$ sec unless otherwise mentioned.

- Next, we consider the parameters related to swimming. We set the steady swimming speed to $v_0 = 2$ BL/s, because many species of fish take the steady swimming speed ∼1.5–3.0 BL/s [24–26]. It is reasonable that we define the characteristic timescale of steady swimming τ_0 from the relation between the typical velocity v_0 and acceleration of fish [18]. The acceleration is on the order of 0.1–1 BL/s^2 when the fish swims steadily [27, 28], from which we estimated $\tau_0 = 1$ s [18]. The self-propelled force is approximated as $C(v_0^2 - v_i^2) \simeq 2Cv_0(v_0 - v_i)$ for steady swimming ($v_i \simeq v_0$), and therefore the characteristic timescale of steady swimming is $1/(2Cv_0)$ from the equation of motion Eq. (5). Therefore, identifying $1/(2Cv_0)$ with τ_0, we obtain $C = 1/(2v_0\tau_0) = 0.25$ BL^{-1}. Note that C can be also estimated from the drag coefficient, $C = \rho_w C_D S/m$, where $\rho_w \simeq 1000$ kg m^{-3} is the water density, $C_D \gtrsim 0.01$ is the drag coefficient of fish at the Reynolds number $\sim 10^5$ [29], $S \lesssim l_b h_b$ is the lateral surface area of the body, and m is the body mass. The mass is estimated as $m = \rho' l_b^2 h_b$ ($\rho' \sim 41$ kg m^{-3}) for the average of many species of fish [23], and then we obtain $C \sim \rho_w C_D/(\rho' l_b) \sim \mathcal{O}(0.1)$ BL^{-1}. The coefficients of noises D_v and D_θ are the control parameters. We mostly use $D_\theta = 0.01$, for which the swing angle by the noise per unit time is $\sqrt{2D_\theta} \sim 8^\circ$.

- There are several parameters related to the forces. We read the characteristic interaction ranges r_e, ρ_e, r_o, and r_a from the experiments. A golden shiner has the equilibrium distance for front-back $r_e \sim 2$ BL, the equilibrium distance for left-right $\rho_e \sim 1$ BL, and the distance of maximum attraction $r_a \sim 3$ BL [20]. Note that $r_a \sim 3$ BL is close to the limiting distance for detecting the pattern of neighbors [15]. In the case of mosquitofish (*Gambusia holbrooki*), $r_e \sim 2$ BL and $r_a \sim$ 3–5 BL [21]. For rummy-nose tetra (*Hemigrammus rhodostomus*), $\rho_e \sim 1$ BL, $r_a \sim$ 6–7 BL, and the distance of maximum alignment is $r_o \sim 3$ BL [22]. Because r_o is between ρ_e and r_a, we chose $r_o = 2$ BL in our model. We set the characteristic length of alignment to $l_o = 2$ BL, because the alignment force at zero relative distance is about half of the peak value [22].

- The amplitudes of the forces and the angular velocities f_r, f_a, ω_r, ω_a, and ω_o are determined as follows. (Note that a body mass is rescaled as 1 and then the unit of force is the same as that of acceleration BL s^{-2}.) If the neighbor is in front of the focal agent i with an almost zero distance, it is reasonable that the speed v_i damps to zero. The balance between self-propulsion and repulsive speeding force gives $f_r \sim v_0/\tau_0 = 2.0$ BL s^{-2}. The attractive speeding force f_a is about three times as large as f_r [20, 21], and thus $f_a = 6.0$ BL s^{-2}. We set the attractive angular velocity to $\omega_a = 3.0$ s^{-1} considering the characteristic speed v_0 and the relation $\omega_a \sim f_a/v_0$. For the repulsive angular velocity ω_r, the contribution of the repulsive turning force is relatively small compared with the repulsive speeding force [20, 21], and we read $\omega_r \lesssim \omega_a/3.5$ from the experimental data [22] and set $\omega_r = 0.5$ s^{-1}($< f_r/v_0$). The aligning angular velocity ω_o is the control parameter, but ω_o can be estimated as about half of ω_a from the experiment [22]. Note that f_a and ω_a are directly evaluated by the experimental data. Rummy-nose tetra shows burst swimming for a period of 0.1-0.2 s and the speed increases with 1-2 BL/s [22], and therefore the acceleration is 5-10 BL s^{-2}. Moreover, the burst swimming of salmon and trout continues for ∼1-2 s and the speed increases with 6-10 BL/s [24], and then the acceleration is 3-10 BL s^{-2}. This is consistent with our choice of the parameter $f_a = 6.0$ BL s^{-2}. In addition, Rummy-nose tetra changes the angle of the direction of motion by $\sim 0.35^\circ$ during burst swimming [22], and then we obtain $\omega_a \sim$ 2-3 s^{-1}.

TABLE S2. List of the parameters. The column "experiment" shows the values directly taken from the experiments, and "estimation" corresponds to the estimation by the method mentioned in the *SI Text.* We rescaled the parameter values by the body length (BL) l_b, timescale $\tau_0 = 1$sec, and body mass m. Regarding "subcontrol parameters", we used the values mentioned as "mostly" unless otherwise stated.

symbol	definition	experiment	estimation	simulation (rescaled)
h_b	body height	$\sim$ 0.3 BL [23]	–	0.3
l_e	position of eye from center of body	–	$\lesssim 0.5$	0.4
N_b	number of visual bin	–	$\gtrsim \mathcal{O}(10^2)$	720
$\delta_b^{\parallel}$	width of a bin	–	related to N_b	0.5°
r_0	limiting resolution distance	16-39 BL [15]	–	$\simeq 28.6$
$\delta_0^{\parallel}$	horizontal resolution angle	–	related to r_0	2°
κ	sharpness of signal	–	–	subcontrol parameter (mostly 2.5)
χ	heterogeneity of density of ganglion cell	–	$\sim 1/3$	control parameter $\in [0,1]$
$\hat{A}$	reference amplitude of signal	–	–	1.0
β	dependence of relative speed	–	–	subcontrol parameter (mostly 4.0)
τ_ϕ	time scale for ϕ_i	–	$\sim$ 1 s	subcontrol parameter (mostly 1.0)
v_0	steady swimming speed	$\sim$1.5-3.0 BL/s [24–26]	–	2.0
C	coefficient of Newton's drag	–	$\sim$ 0.25 BL^{-1}	0.25
D_v	diffusion coefficient for v_i	–	–	subcontrol parameter (mostly 0.01)
D_θ	diffusion coefficient for θ_i	–	–	subcontrol parameter (mostly 0.01)
r_e	equilibrium distance for front-back	$\sim$ 2 BL [20, 21]	–	2.0
ρ_e	equilibrium distance for left-right	$\sim$ 1 BL [20, 22]	–	1.0
r_a	distance of maximum attraction<br>distance of detection of pattern	$\sim$ 3-7 BL [15, 20–22]	–	3.0
r_o	distance of maximum alignment	$\sim$ 3 BL [22]	–	2.0
l_o	characteristic length of alignment	–	$\sim$ 2.0 BL	2.0
f_r	repulsive speeding force	–	$\sim$ 2.0 BL s^{-2}	2.0
f_a	attractive speeding force	–	$\sim$ 6.0 BL s^{-2}	6.0
ω_r	repulsive angular velocity	–	$\lesssim$ 0.8 s^{-1}	0.5
ω_a	attractive angular velocity	–	$\sim$ 3.0 s^{-1}	3.0
ω_o	aligning angular velocity	–	$\sim$ 1.5 s^{-1}	control parameter $\in [0,3]$

D. Numerical integration of equation

We numerically integrated Eqs. (4), (5), and (6) by the Euler method with the time step $\Delta t = 0.005$. For the stochastic part, we use Itô integral and replace the standard Wiener process by $\xi\sqrt{\Delta t}$, where ξ is a random number generated by the standard normal distribution.

II. BEHAVIOR OF SELECTIVE DECISION MAKING

A. Definition of probability distribution

Here, we consider the situation in which the focal agent follows the virtual agents that have the mutual distance L (see Figs. 2(a),(b)). We define the probability distribution of the position of the focal agent $P(x, y, L)$ as follows. In a simulation, we count the event in which a focal agent is located at a spatial bin (x, y) in the coordinate system moving with the virtual agents. The area of measurement is $x \in [-15, 15], y \in [-4, 4]$, and the width of a bin is $\Delta x = \Delta y = 0.25$. We performed 1000 simulations for each parameter set, and normalized the probability distribution by $\int_{-15}^{15} \mathrm{d}x \int_{-4}^{4} \mathrm{d}y\, P(x, y; L) = 1$. We started measurement at $t = 5$ to ensure that $P(x, y; L)$ does not include the initial state, and took the time average over $t \in [5, 55]$ and ensemble average. Note that our aim is to observe the selective behavior in the steady state ($t \gtrsim 5$), similar to those in Refs. [9, 11], and not in observation of the initial attraction to a virtual agent ($t \lesssim 5$). The marginal probability distribution $P(x; L)$ is defined as the integral of $P(x, y, L)$ over the rear side of the virtual agents:

$$P(x; L) = \frac{\int_{-4}^{0} \mathrm{d}y P(x, y; L)}{\int_{-15}^{15} \mathrm{d}x \int_{-4}^{0} \mathrm{d}y P(x, y; L)}. \tag{S13}$$

The distance L is incremented by the step $\Delta L = 0.05$.

B. Parameter dependence and asymmetrical configuration

Let us discuss the parameter dependence of the bifurcation process. In the main text, we used the parameters $\chi = 0.3$ and $\omega_o = 1.0$, for which the vortex pattern emerges for 100 agents. Figs. S6(a), S7(a) are the enlarged maps of $P(x; L)$ in Figs. 2(c),(d), respectively. We confirmed that the bifurcation process is little affected by shifting the initial position of the focal agent closer to the virtual agents, from $y_{\mathrm{f}} = -r_a$ to $y_{\mathrm{f}} = -0.5r_a$ (see Fig. S7(b)).

We changed the sharpness parameter κ of the signal from $\kappa = 2.5$ in the main text. For $\kappa = 0.1$, the bifurcation does not occur as shown in Figs. S6(b), S7(c). This is because the signals from the virtual agents are widely overlapped and the focal agent cannot focus attention to a single virtual agent. On the other hand, for $\kappa = 3.5$, the bifurcation structure becomes slightly clearer than the case $\kappa = 2.5$ (see Figs. S6(c), S7(d)).

The noise coefficients D_v and D_θ are changed from $D_v = D_\theta = 0.01$ in the main text. The bifurcation process did not change qualitatively for both weak noises $D_v = D_\theta = 0.001$ and strong noises $D_v = D_\theta = 0.02$. However, for $L \sim 10$ with three virtual agents, a focal agent is attracted more frequently to the left and right virtual agents for weak noises, and is equally attracted to the three virtual agents for strong noises (see Figs. S6(d),(e), S7(e),(f)). We also studied the bifurcation for $\chi = 0$ and $\omega_o = 3.0$, which give rise to a polarized school for 100 agents. In this case, the focal agent tends to be located at the side of the each virtual agent due to the alignment interaction, but the qualitative feature of the bifurcation structure does not change (see Figs. S6(f), S7(g)).

Next, we consider asymmetrical configuration with three virtual agents (see Fig. S8(a)), with the center virtual agent v3 located at $x_{\mathrm{v3}} = L_{\mathrm{asym}} \in [0, L]$. Note that the limit $L_{\mathrm{asym}} \to L$ corresponds to the two virtual agents system due to the overlap of v2 and v3. Figs. S8(b)-(f) show the marginal distribution $P(x; L, L_{\mathrm{asym}})$ as a function of L_{asym}, where the increment is $\Delta L_{\mathrm{asym}} = 0.05$. When $L_{\mathrm{asym}} = 0$, the cases $L = 2.0$ and $L = 2.5$ result in a two-way fork, $L = 4.0$ and $L = 5.0$ to a three-way fork, and $L = 3.0$ is intermediate (see also Fig. 2(d)). For any L, the branch of the fork closest to v3 moves with v3, and the map converges to a two-way fork for two virtual agents with $L_{\mathrm{asym}} \to L$. In particular, for $L \gtrsim 3.0$, the probability is peaked at the center between v2 and v3, and the probability near v1 almost vanishes for an intermediate value of L_{asym}. Fig. S8(g) shows the change of $P(x; L, L_{\mathrm{asym}})$ with $L = 3.0$ as a function of $L_{\mathrm{asym}} \in [0, 1]$. For $L_{\mathrm{asym}} = 0$, $P(x; L, L_{\mathrm{asym}})$ has symmetrical four peaks. As we increase L_{asym}, the right two peaks are united, the leftmost peak diminishes, and the second peak from the left moves with the position of v3. We chose $L = 3.0$ because the three-way fork bifurcation starts at $L = 3.0$ in our model, and because the asymmetrical configuration for zebrafish was studied at the distance where the three way fork bifurcation starts (see Fig. 3(F) and

Fig. 4(B) in Ref. [9]): the body length of zebrafish is 1 ± 0.1 cm, and the three-way fork bifurcation started at $L \sim 6$ cm, and the asymmetrical configuration was studied for $L = 6$ cm.

C. Conventional particle model

For comparison with our visual model, we considered a conventional particle model. It uses a point particle as an agent, and the force is averaged over all the neighbors (virtual agents). The equations of motion of the focal agent f read

$$\frac{\mathrm{d}v_{\mathrm{f}}}{\mathrm{d}t} = C(v_0^2 - v_{\mathrm{f}}^2) + \frac{1}{N_{\mathrm{v}}}\sum_{\mathrm{n}=1}^{N_{\mathrm{v}}} F(\phi_{\mathrm{f,vn}}, r_{\mathrm{f,vn}}) + \eta_{v,\mathrm{f}} \tag{S14}$$

and

$$\frac{\mathrm{d}\theta_{\mathrm{f}}}{\mathrm{d}t} = \frac{1}{N_{\mathrm{v}}}\sum_{\mathrm{n}=1}^{N_{\mathrm{v}}} \Omega(\phi_{\mathrm{f,vn}}, r_{\mathrm{f,vn}}, \psi_{\mathrm{f,vn}}) + \eta_{\theta,\mathrm{f}}, \tag{S15}$$

where $\mathrm{n} = 1, \cdots, N_{\mathrm{v}}$ is the index of the virtual agents, $r_{\mathrm{f,vn}} = |\boldsymbol{r}_{\mathrm{vn}} - \boldsymbol{r}_{\mathrm{f}}|$ is the distance between the focal agent and the n-*th* virtual agent (vn), $\phi_{\mathrm{f,vn}}$ is the relative angle of position of vn from f, and $\psi_{\mathrm{f,vn}}$ is the relative heading angle between vn and f. The functions F and Ω are the same as that of our model (see Eqs. (11),(13)). In other words, the force average on the direction of visual attention $\langle \cdots \rangle_\mu$ in our model is replaced by the average over all virtual agents $\frac{1}{N_{\mathrm{v}}}\sum_{\mathrm{n}=1}^{N_{\mathrm{v}}} \cdots$ in the conventional particle model.

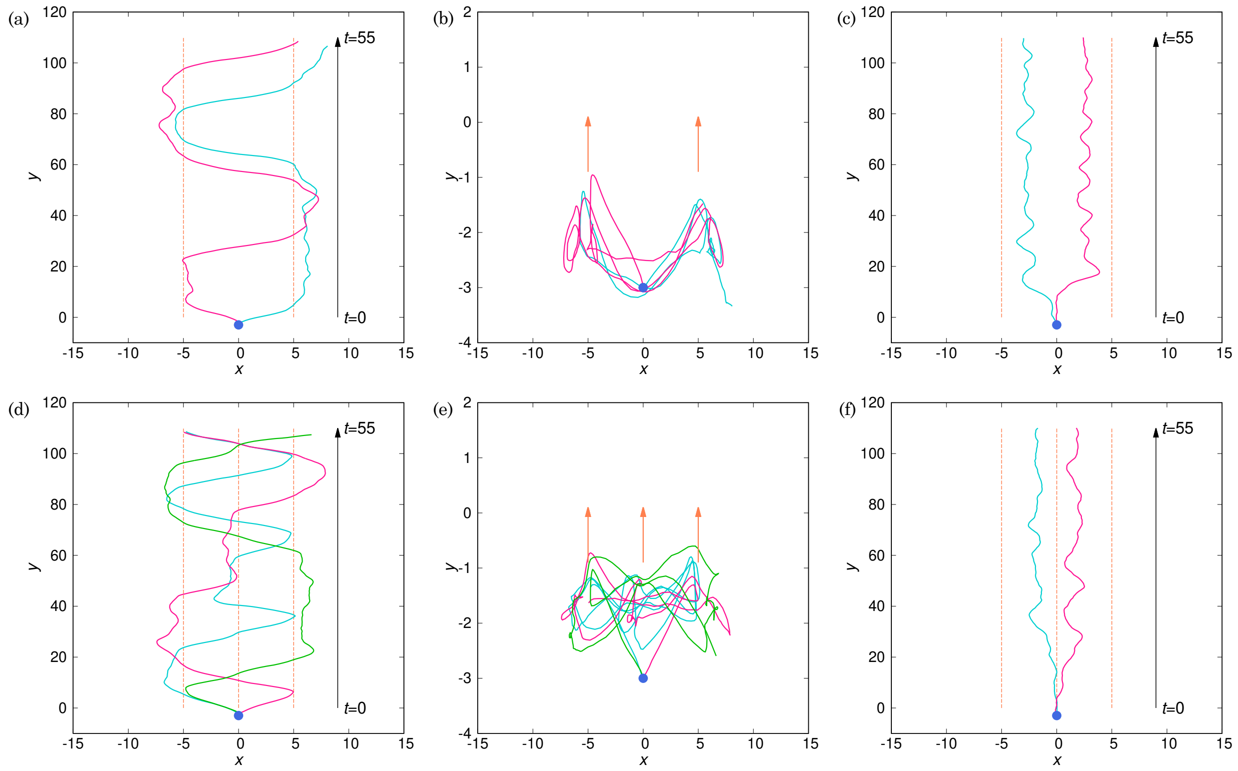

FIG. S3. The examples of the trajectory of the focal agent with (a)-(c) two virtual agents with $L = 10$ and (d)-(f) three virtual agents with $L = 5$ during $t \in [0, 55]$: (a),(b),(d),(e) our model with $\chi = 0.3, \omega_o = 1.0$, and (c),(f) the conventional particle model. The initial position of the focal agent is shown by the blue point, and the trajectories are the cyan, pink, and green solid lines. In (a),(c),(d) and (f), we show the trajectory in the laboratory frame and the orange dashed lines represent the trajectory of the virtual agents. In (b) and (e), we use the comoving frame of the virtual agents (moving along the y-axis with the speed v_0), and the orange arrows represent the virtual agents.

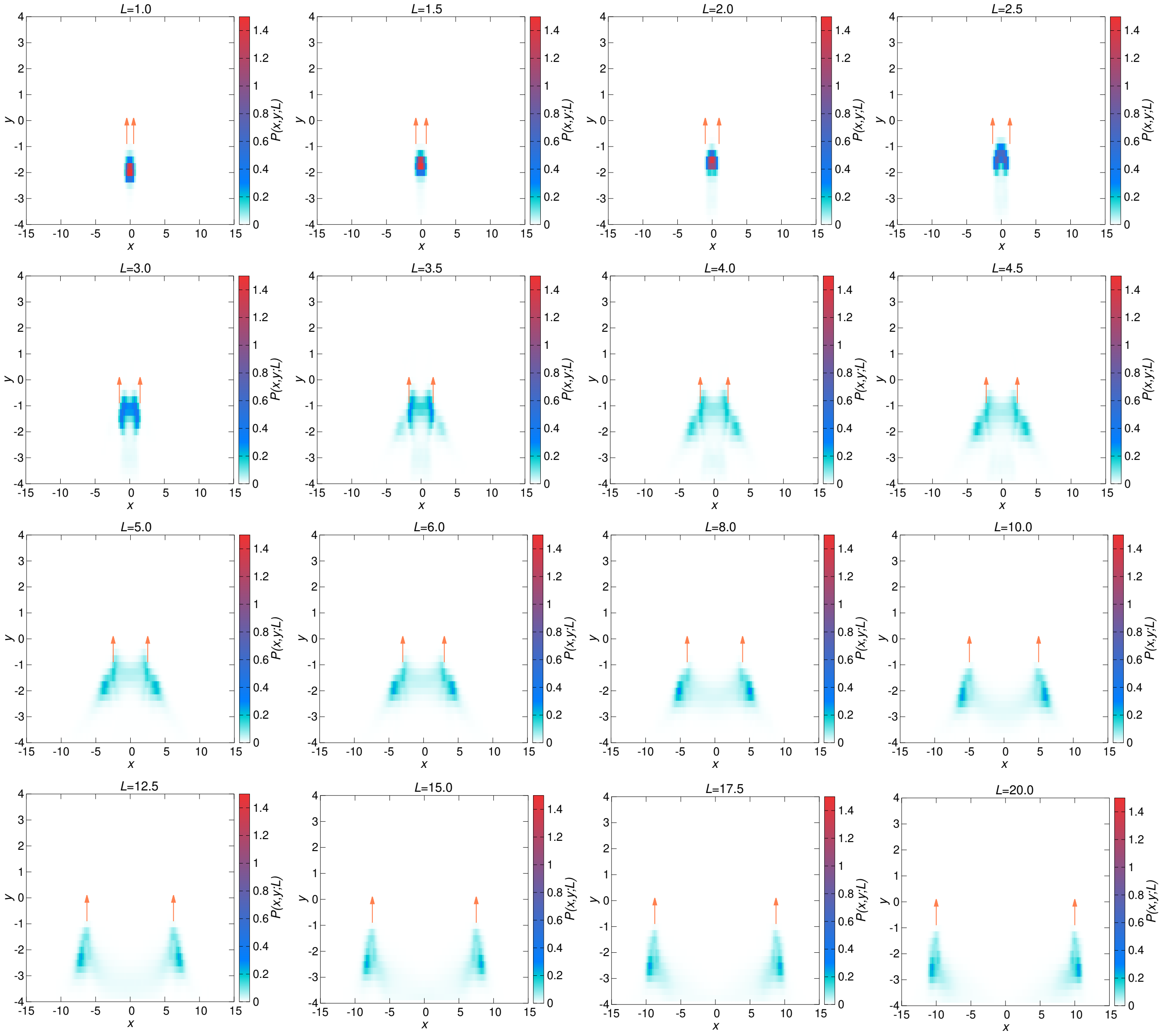

FIG. S4. The L-dependence of the probability distribution $P(x, y; L)$ for two virtual agents with $\chi = 0.3$ and $\omega_o = 1.0$. The orange arrows represent the virtual agents. We changed L from 1.0 (top left) to 20.0 (bottom right).

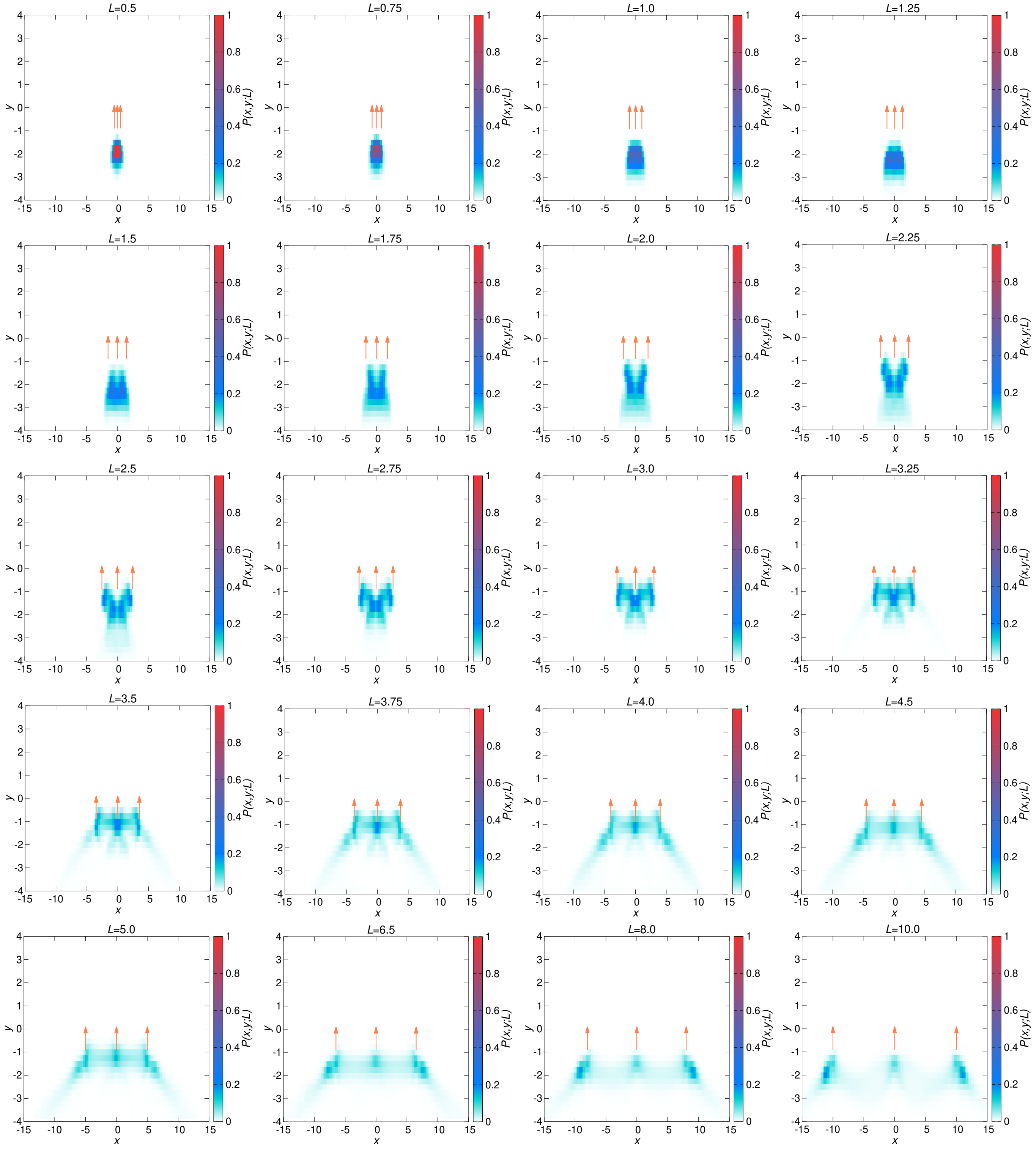

FIG. S5. The L-dependence of $P(x, y; L)$ for three virtual agents with $\chi = 0.3$ and $\omega_o = 1.0$. The orange arrows represent the virtual agents. We changed L from 0.5 (top left) to 10.0 (bottom right).

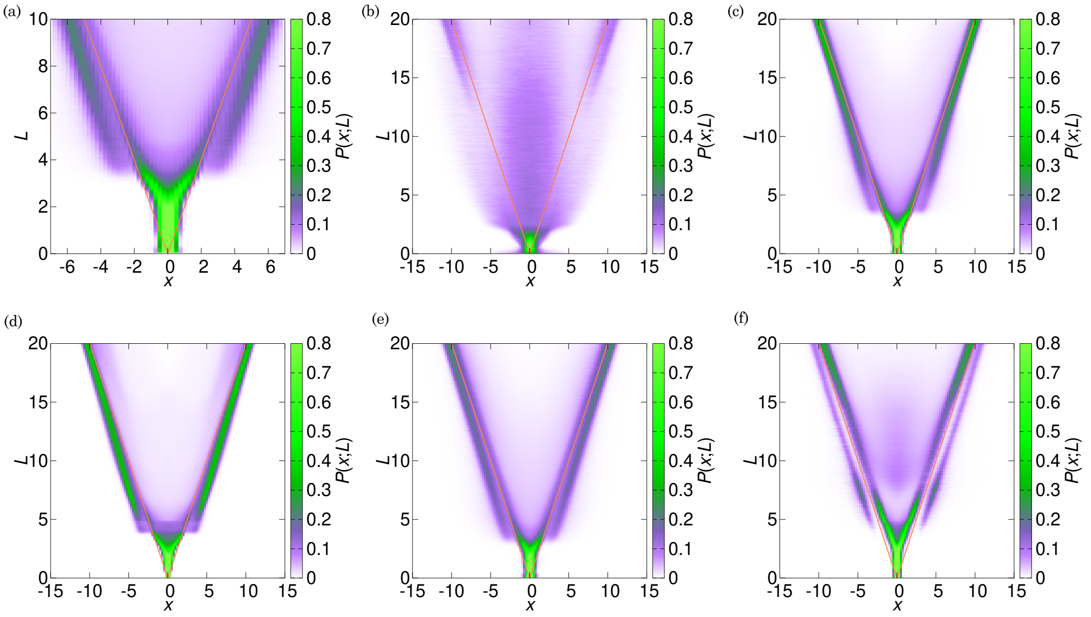

FIG. S6. The marginal probability distribution $P(x; L)$ for two virtual agents. The orange lines represent the x-coordinate of the virtual agents. (a) Enlarged map ($L \leq 10$) of Fig. 2(c) for the standard parameter set $\chi = 0.3$, $\omega_o = 1.0$, $\kappa = 2.5$, and $D_v = D_\theta = 0.01$, which are changed to (b) $\kappa = 0.1$, (c) $\kappa = 3.5$, (d) $D_v = D_\theta = 0.001$, (e) $D_v = D_\theta = 0.02$, (f) $\chi = 0$, $\omega_o = 3.0$, respectively.

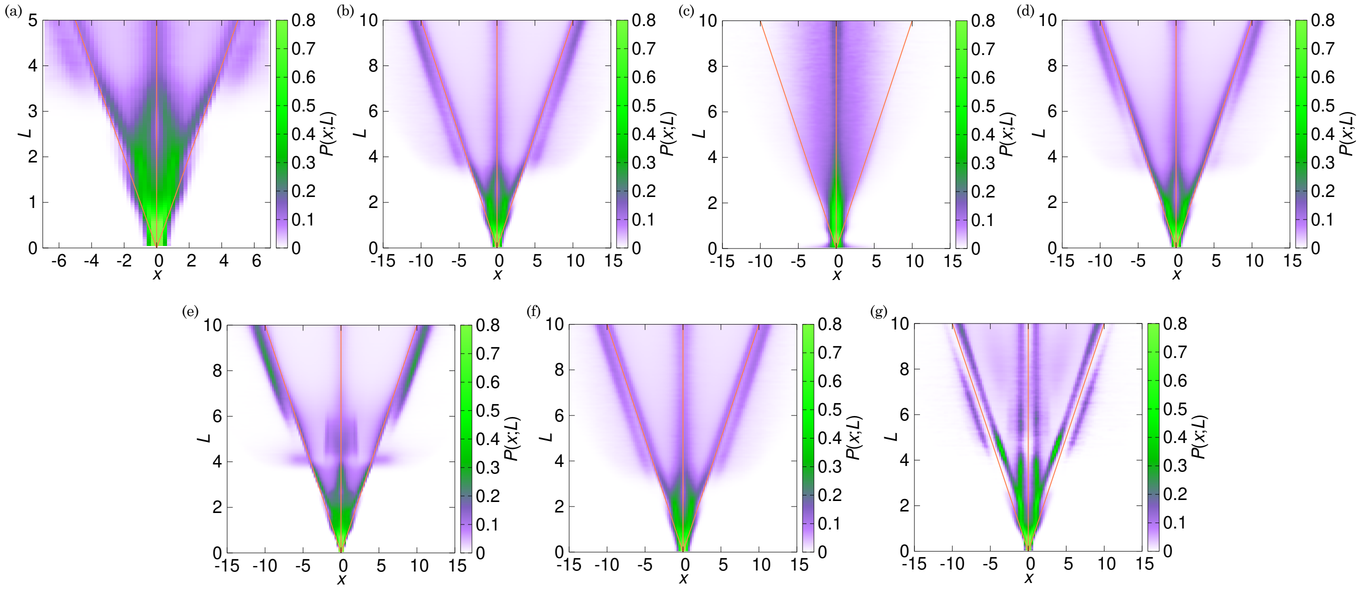

FIG. S7. The marginal probability distribution $P(x; L)$ for three virtual agents. The orange lines represent the x-coordinate of the virtual agents. (a) Enlarged map ($L \leq 5$) of Fig. 2(d) for the standard parameter set $\chi = 0.3$, $\omega_o = 1.0$, $\kappa = 2.5$, $D_v = D_\theta = 0.01$ and the initial position $(x_\mathrm{f}, y_\mathrm{f}) = (0, -r_a)$. They are changed to (b) $(x_\mathrm{f}, y_\mathrm{f}) = (0, -0.5r_a)$, (c) $\kappa = 0.1$, (d) $\kappa = 3.5$, (e) $D_v = D_\theta = 0.001$, (f) $D_v = D_\theta = 0.02$, and (g) $\chi = 0$, $\omega_o = 3.0$, respectively.

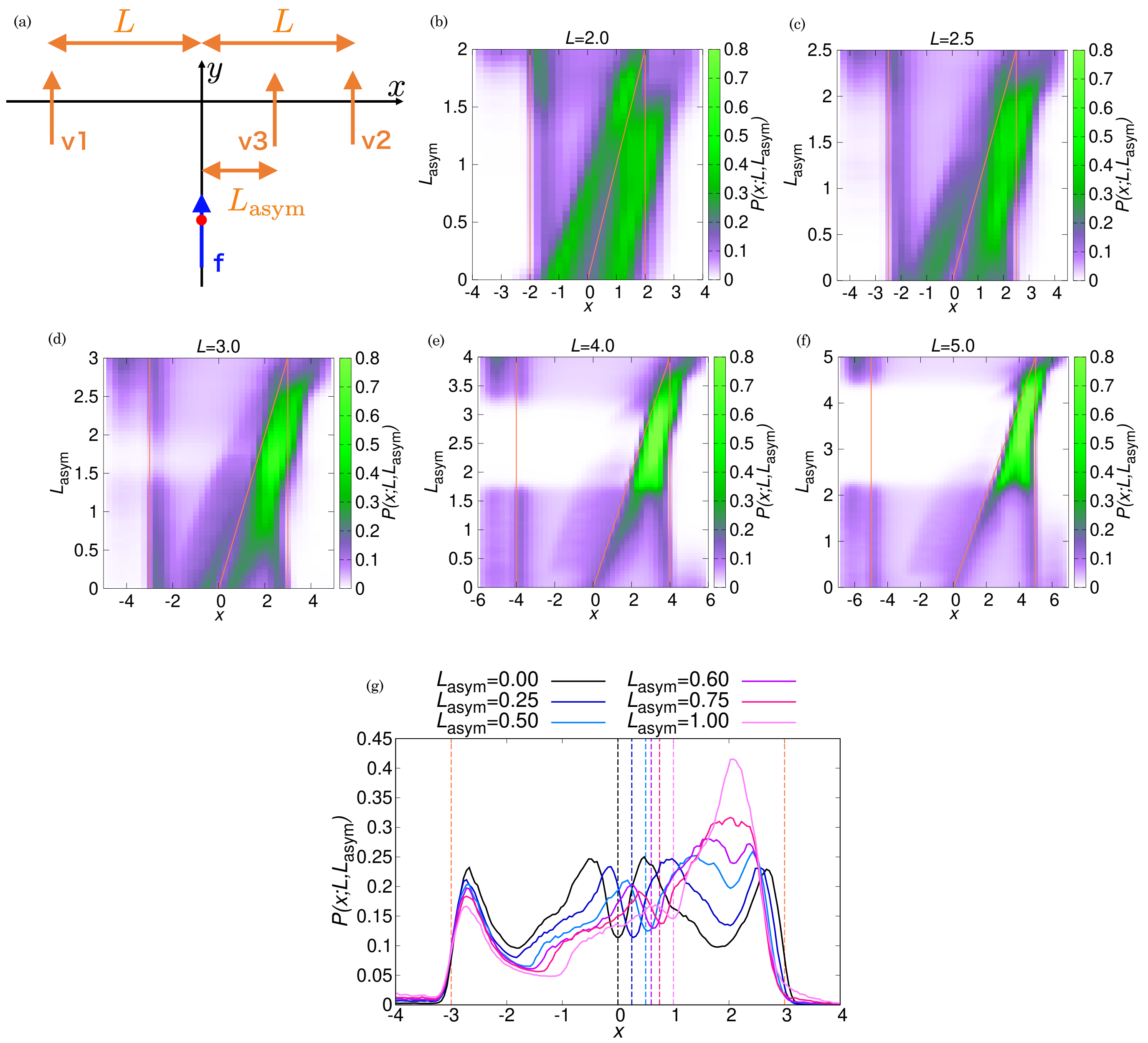

FIG. S8. The marginal probability distribution $P(x; L, L_{\rm asym})$ for the asymmetrical system of three virtual agents with $\chi = 0.3, \omega_o = 1.0$. (a) Schematic illustration of the initial condition. (b)-(f) The marginal probability distribution $P(x; L, L_{\rm asym})$. The orange lines represent the x-coordinate of the virtual agents. (g) $P(x; L, L_{\rm asym})$ for $L = 3.0$ and $L_{\rm asym} \in [0, 1]$. The dashed vertical lines represent the x-coordinates of the virtual agents: the left and the right dashed orange lines correspond to v1 and v2, and the colored dashed lines near the center corresponds to v3 for different values of $L_{\rm asym}$.

III. COLLECTIVE MOTION

For collective motion, we used 100 agents unless otherwise stated.

A. Definition of the splitting

We judged that a splitting occurred when both of the following conditions are met at the end of a simulation.

- Condition 1: The maximum distance of an agent from the center of mass $\boldsymbol{r}_G = \sum_{i=1}^{N} \boldsymbol{r}_i/N$ is larger than $3r_0 \sim 85$.
- Condition 2: The number of agents in the largest cluster is smaller than $0.95N$, where the radius of the clustering method is the limiting distance $r_0 \sim 28$.

The first condition is justified because, if a cluster splits in the middle of a simulation, resultant small clusters are separated from each other with the typical distance $\sim \mathcal{O}(1000)$ at the end of a simulation ($t = 800$). The distance is much larger than the characteristic size of a steady cluster $\sim \mathcal{O}(10)$ (see Fig. 3(a)). We confirmed that a small cluster with a few agents tends to be attracted to the largest cluster. However, the condition 1 is also accidentally satisfied when, for example, two polarized school clusters move in parallel maintaining a small distance. The condition 2 is added to judge such a case as a splitting.

B. Cluster size and duration of the order

We took the average of various quantities $Q(t)$ over time and using an ensemble of 25 simulations. If the cluster is not splitting in a simulation, we took the time average $\tilde{Q}$ over the time window $t \in [300, 800]$. For example, we show the time-averaged order parameters (Eqs. (17),(17)) in Fig. S10. We then averaged $\tilde{Q}$ over simulations in which a splitting did not occur to get the ensemble average Q. For example, the phase diagram (Fig. 3(b)) is drawn by using the ensemble-averaged order parameters P and M. The standard deviation of Q is defined as the square root of the variance of $\tilde{Q}$ in no-splitting simulations.

The size of the cluster is measured as follows. The radius of gyration R_g is defined as the average of

$$R_g(t) = \sqrt{\frac{1}{N}\sum_{i}^{N} \boldsymbol{c}_i^2(t)}, \tag{S16}$$

where $\boldsymbol{c}_i(t) = \boldsymbol{r}_i(t) - \boldsymbol{r}_G(t)$ is the relative position from the center of mass. The moment of inertia tensor is

$$I_{ab} = \sum_i (\boldsymbol{c}_i^2 \delta_{ab} - c_{i,a} c_{i,b}), \tag{S17}$$

where $a, b = x, y$ and δ_{ab} is the Kronecker delta. Its eigenvalues I_1 and I_2 are the principal momenta of the inertia,

$$I_1 = \frac{\operatorname{tr} I + \sqrt{(\operatorname{tr} I)^2 - 4\det I}}{2}, \; I_2 = \frac{\operatorname{tr} I - \sqrt{(\operatorname{tr} I)^2 - 4\det I}}{2}. \tag{S18}$$

The corresponding eigenvectors $\boldsymbol{u}_\alpha$ ($\alpha = 1, 2$) are obtained as $u_{\alpha,x} = I_{xy}$, $u_{\alpha,y} = I_\alpha - I_{xx}$ by solving

$$\begin{bmatrix} I_{xx} - I_\alpha & I_{xy} \\ I_{xy} & I_{yy} - I_\alpha \end{bmatrix} \begin{bmatrix} u_{\alpha,x} \\ u_{\alpha,y} \end{bmatrix} = 0, \tag{S19}$$

and then normalized as $\hat{\boldsymbol{u}}_\alpha$. We define the cluster's length in the direction of $\hat{\boldsymbol{u}}_\alpha$ as

$$S_\alpha(t) = \frac{1}{N}\sum_{i=1}^{N} |\boldsymbol{c}_i(t) \cdot \hat{\boldsymbol{u}}_\alpha(t)|, \tag{S20}$$

and the aspect ratio S_r as the average of

$$S_r(t) = S_2(t)/S_1(t). \tag{S21}$$

Note that $S_r > 1$ because $I_1 > I_2$ and $S_1 < S_2$.

We measured the duration of the polar and rotational order $\tilde{T}_P$ and $\tilde{T}_M$ by the time that $P(t)$ (Eq.(17)) and $M(t)$ (Eq.(17)) exceed the threshold values $P_{\rm th} = 0.8$ and $M_{\rm th} = 0.5$ in $t \in [300, 800]$, respectively (see also Fig. S10). We normalized $\tilde{T}_P$ and $\tilde{T}_M$ by the duration of measurement so that they are ranged in [0, 1], and then obtained their ensemble averages T_P and T_M using only no-splitting cases. From Fig. S11(a),(b), we confirm that a vortex has a large T_M, a polarized school and turning has a large T_P, and a swarm is characterized by small T_P and T_M. Thus the order parameters of the patterns (i)-(iv) do not show a large fluctuation if the cluster does not split.

C. Dependence on the initial condition

We examined the initial condition dependence of the collective motion as follows. First, we counted the frequency of splitting in 25 simulations and defined the splitting ratio p_s as the ratio of the number of splitting simulations in 25 simulations. As shown in Fig. S11(c), the splitting frequently occur for polarized school and turning, in addition to unsteady aggregation.

To see whether the splitting for the polarized school and the turning occurs due to the random initial configuration, we tested the aligned initial condition. We positioned 100 agents randomly in a circle of radius 7, with the same orientation $\theta_i = 0$ and speed $v_i = v_0$ but with the random angle of visual attention $\phi_i \in [-\pi, \pi]$. Therefore, all agents move in the positive direction of x-axis at $t = 0$. As shown in Fig. S12(a), the aligned initial condition reduced p_s compared to the random initial condition, for $\omega_o = 3.0$ and $\chi \in [0, 1]$. In particular, a splitting seldom occurred for a polarized school ($\chi \sim 0$). Thus we may conclude that the splitting for a polarized school is due to the randomness of the initial orientation and that the polarized school becomes stable once the direction of motion is aligned. On the other hand, for moderate or large χ, a splitting can occur also because a cluster turns from its frontal edge, which reflects the large aspect ratio S_r for $\chi = 0.2$–1.0 as shown in Fig. S12(a).

We studied the effect of the randomness of the initial orientation in the polarized school for $\chi = 0$, $\omega_o = 3.0$ in more detail. For the initial condition, we randomly set the orientation of the agents within $\theta_i \in [-\delta\theta/2, \delta\theta/2]$, where $\delta\theta$ is a constant. As shown in Fig. S12(b), the polarized school is stable up to $\delta\theta = \pi$. Note that $\delta\theta = \pi$ is larger than the typical angular deviation in the polarized school, $\Theta \sim 20^\circ = \pi/9$. The latter was measured using the time autocorrelation function of the orientation of the agent, defined by

$$\Theta(\delta t) = \frac{180}{\pi}\sqrt{\frac{1}{N}\sum_{i=1}^{N}(\delta\vartheta_i(t+\delta t))^2}, \quad \delta\vartheta_i(t+\delta t) = \{\theta_i(t+\delta t) - \theta_P(t+\delta t)\} - \{\theta_i(t) - \theta_P(t)\}. \tag{S22}$$

and shown in Fig. S13. Here, $\theta_P(t)$ is the angle of the polar order parameter vector $\boldsymbol{P}(t) = \frac{1}{N}\sum_{i=1}^{N}\boldsymbol{e}_i(t)$ and δt is the elapsed time.

D. Parameter dependence of the collective pattern

The patterns of collective motion depend not only on χ and ω_o but also on the other parameters. Here we mainly treat (i) the vortex ($\chi = 0.3, \omega_o = 1.0$) as the reference pattern. As shown in Fig. S14(a)-(c), the parameters $\kappa = 2.5$, $\beta = 4.0$, $\tau_\phi = 1.0$ that are mainly used in this paper give a large rotational order parameter M and a small splitting ratio p_s. This result can be interpreted in terms of the relation between the splitting and the visual signal as follows. First, if the signal is shallow or the motion of visual attention is slow, the motion of visual attention cannot keep up with the motion of a neighbor, and then the initial cluster will be dispersed. This situation occurs for small κ (the sharpness of a signal) and large τ_ϕ (the characteristic timescale of visual attention). Second, if the signals from a

neighbor are too deep, the visual attention is fixed at a certain neighbor and does not move to another, and then the initial cluster will be splitted into many small clusters and dispersed. This situation occurs for large κ, small β (the dependence on the relative speed of a signal), and small τ_ϕ. In addition, we study the effect of the noises D_v and D_θ (see Fig. S14(d),(e)). The main effect is that the rotational order parameter M decreases as D_θ increases. The noises have only a minor effect on the splitting ratio p_s.

Finally, we consider the dependence on the number of the agents N. For the initial condition, the radius of the circle is set as $7 \times \sqrt{N/100}$ so that the initial number density is the same as that of the 100 agents case. We use the random initial condition and the aligned initial condition for (i) the vortex ($\chi = 0.3, \omega_o = 1.0$) and (ii) polarized school ($\chi = 0, \omega_o = 3.0$), respectively. As shown in Fig. S15(a),(b), the splitting is prevented for $50 \lesssim N \lesssim 200$ for both the vortex and polarized school. On the other hand, the splitting frequently occurs both for a small number of agents ($4 \lesssim N \lesssim 50$) and a large number ($N \gtrsim 250$). See Discussion Section in the main text for interpretation of this result. In addition, the radius of gyration R_g increases as N increases to $N \gtrsim 90$, but R_g is almost constant for $N \lesssim 90$ (see Fig. S15(c)).

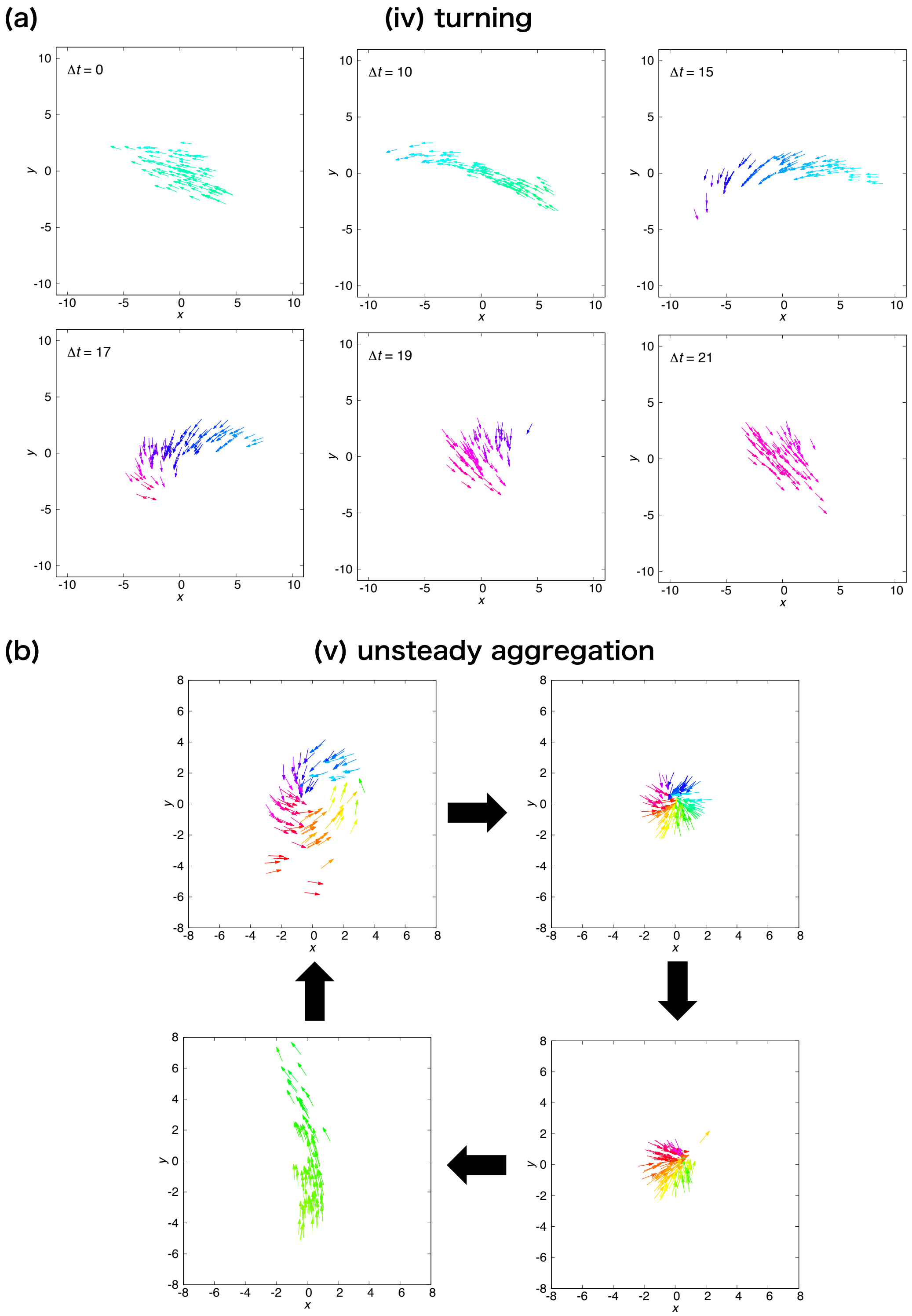

FIG. S9. Snapshots of 100 agents for (a) turning $\chi = 1.0, \omega_o = 3.0$ and (b) unsteady aggregation $\chi = 0.6, \omega_o = 1.75$; Δt is the elapsed time. In each snapshot, the agents are represented in the same way as in Fig. 3(a). In (a), a polarized school is elongated in the direction of motion for $\Delta t \lesssim 10$, and then turns from the front end for $\Delta t \lesssim 19$, and finally becomes straight again at $\Delta t = 21$. In (b), the agents in the vortex (top left) are oriented inward and get stuck (top right), and are gradually aligned (bottom right). Then the cluster is elongated (bottom left) and forms a vortex again by turning from the front end.

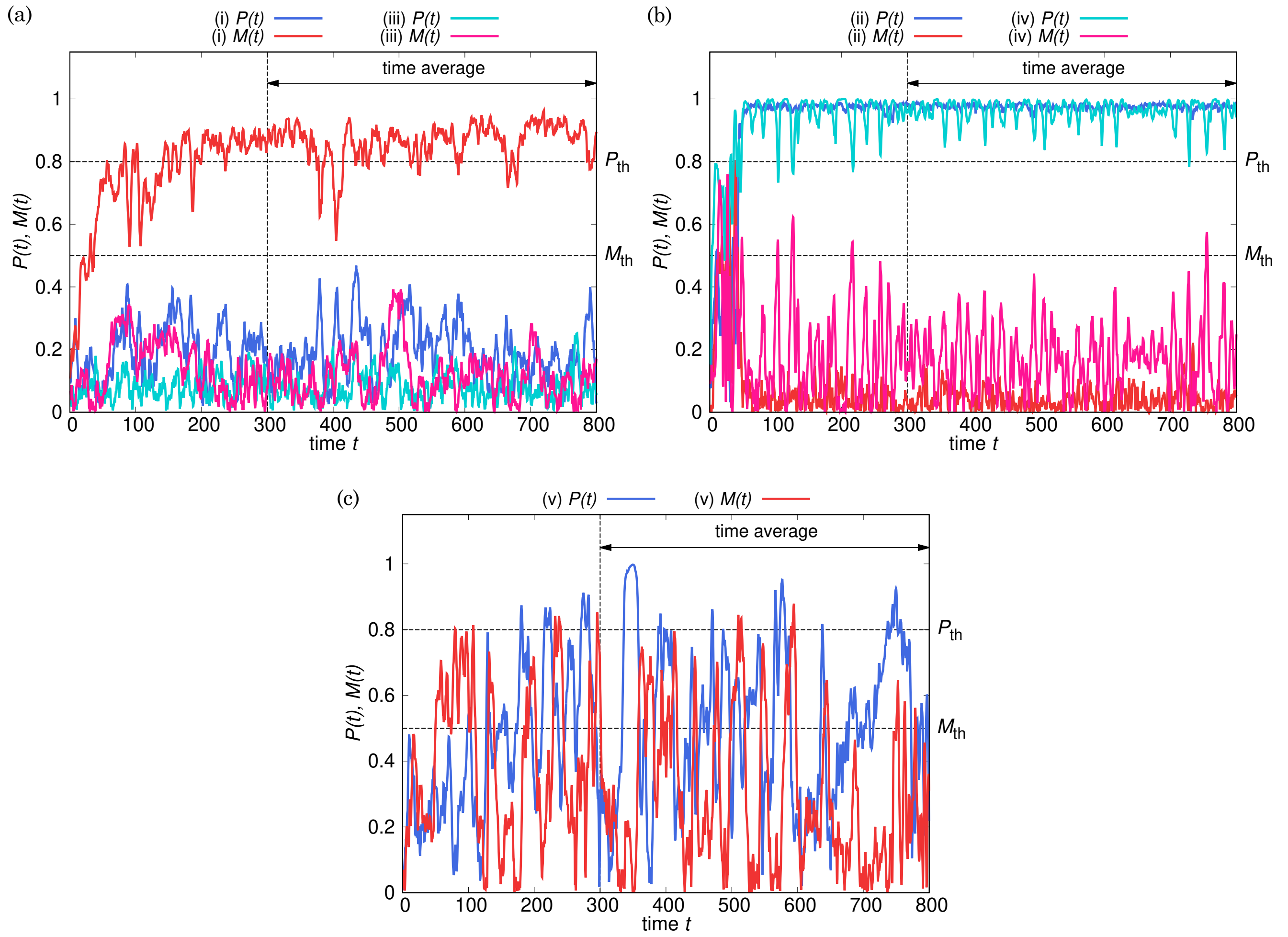

FIG. S10. Time evolution of the order parameters $P(t)$ and $M(t)$ for (a) (i) vortex and (iii) swarm, (b) (ii) polarized school and (iv) turning, and (c) unsteady aggregation. The blue or cyan line shows $P(t)$ and the red or pink line is $M(t)$. The time window $t \in [300, 800]$ corresponds to the interval for time averaging, and $P_{\rm th}, M_{\rm th}$ are the threshold values of P and M (see also *SI Text* and Fig. S11(a),(b)). In (a), the vortex has large values of $M(t)$ and small $P(t)$, but the swarm has small $P(t)$ and $M(t)$. In (b), both the polarized school and the turning have large $P(t)$ and small $M(t)$, and the spikes of $P(t)$ and $M(t)$ describe the turning of the cluster. In (c), the large fluctuations of $P(t)$ and $M(t)$ reflect the vortex-stuck-polarized state loop with irregular time intervals.

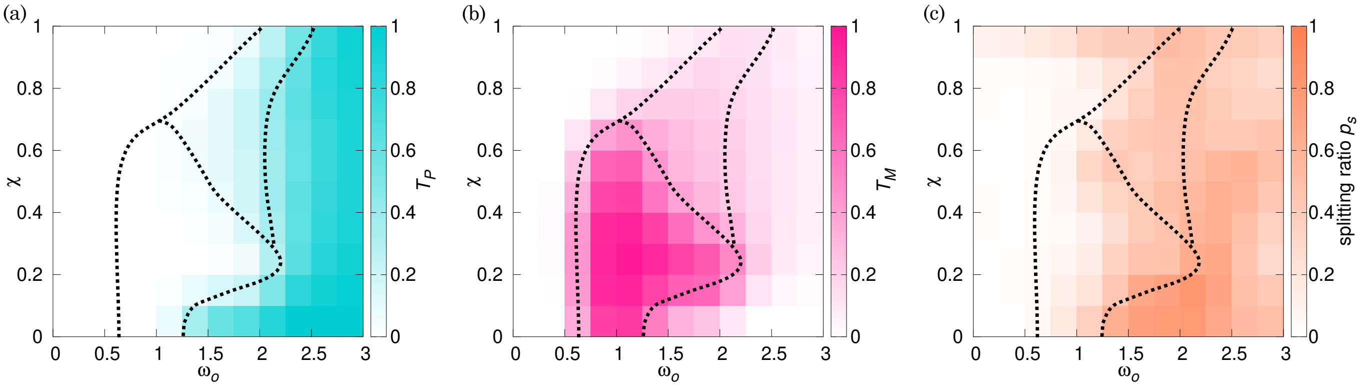

FIG. S11. Maps of (a) the duration of polarization T_P, (b) the duration of rotation T_M, and (c) the splitting ratio p_s. The dashed lines represent the borders between different collective patterns taken from Fig. 3(b).

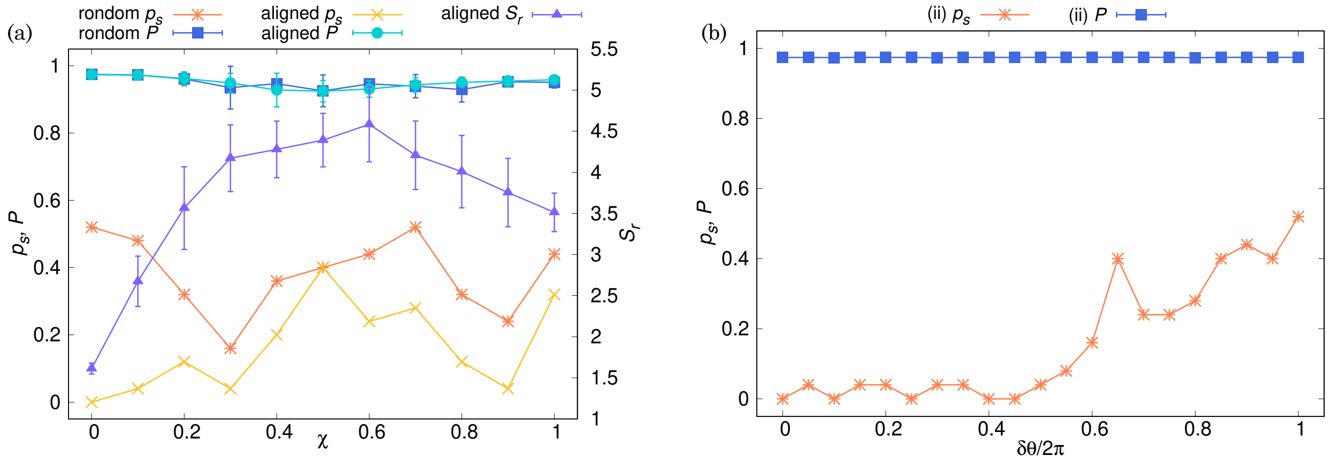

FIG. S12. The relation between the polarized cluster and splitting. The error bars represent the standard deviation. (a) p_s and P (left) and S_r (right) as functions of χ, for $\omega_o = 3.0$. The orange line shows p_s for the random initial condition, and the yellow is p_s for the aligned initial condition. The blue line shows P for the random initial condition, the cyan is P for the aligned initial condition, and the purple is S_r for the aligned initial condition. (b) p_s and P as functions of the initial angle deviation $\delta\theta$ for the polarized school ($\chi = 0, \omega_o = 3.0$). The orange line shows p_s and the blue is P.

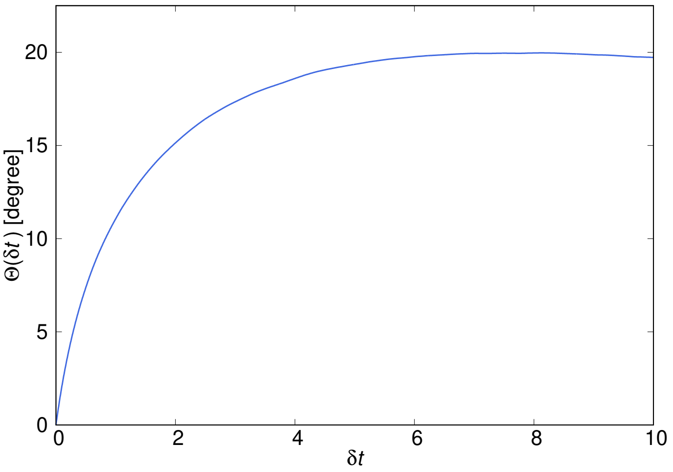

FIG. S13. The time autocorrelation function $\Theta(\delta t)$ of the orientation of the agent for the polarized school ($\chi = 0, \omega_o = 3.0$). The time average is taken over $t \in [300, 20300]$ in a no-splitting simulation. The function is saturated at $\sim 20^\circ$ for the elapsed time $\delta t \gtrsim 5$.

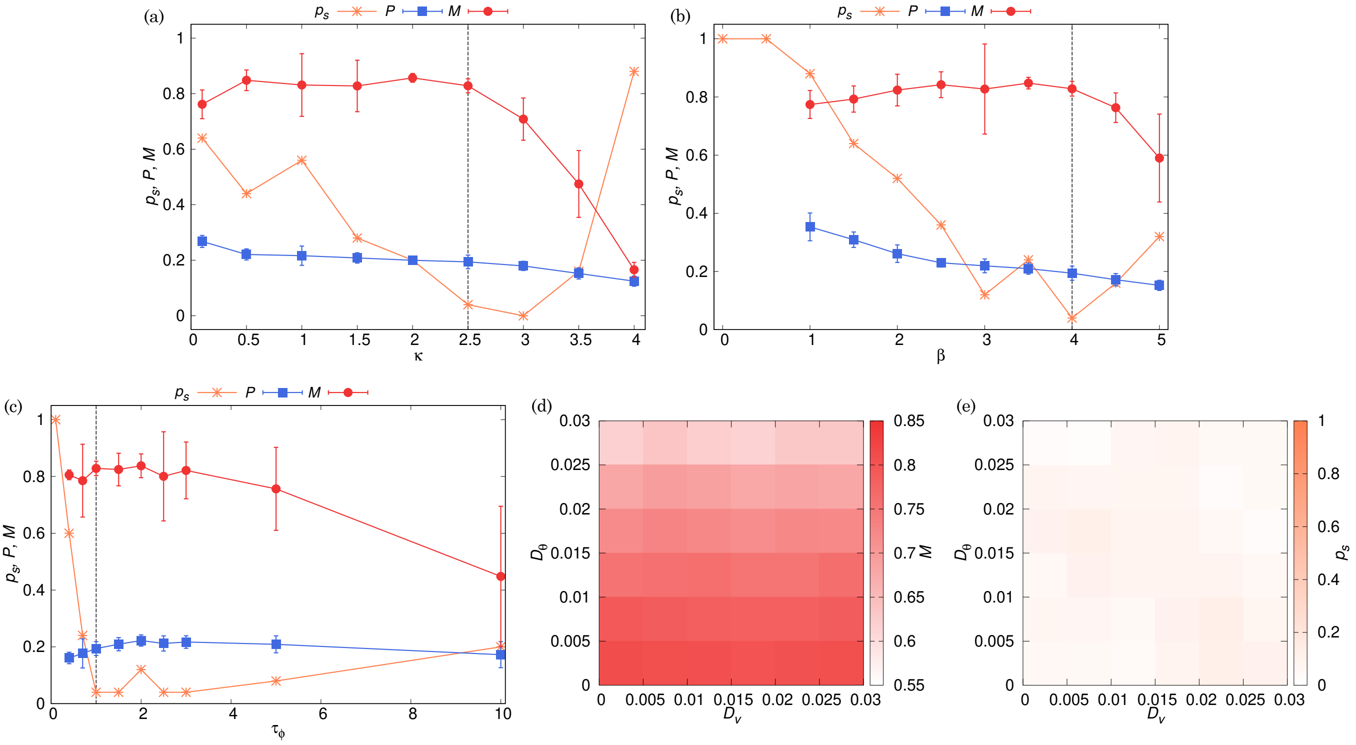

FIG. S14. The parameter dependence of (i) the vortex ($\chi = 0.3, \omega_o = 1.0$). The orange line shows the splitting ratio p_s, the blue is the polar order parameter P, and the red is the rotational order parameter M, as functions of (a) the signal sharpness κ, (b) the dependence on the relative speed of a signal β, (c) the characteristic timescale of the motion of visual attention τ_ϕ. The error bars represent the standard deviation. The dashed black vertical lines represent the parameters that are mainly used in this paper: $\kappa = 2.5$, $\beta = 4.0$, and $\tau_\phi = 1.0$. (P and M are not shown for $p_s = 1$.) Dependence of (d) M and (e) p_s on the noise strengths D_v and D_θ.

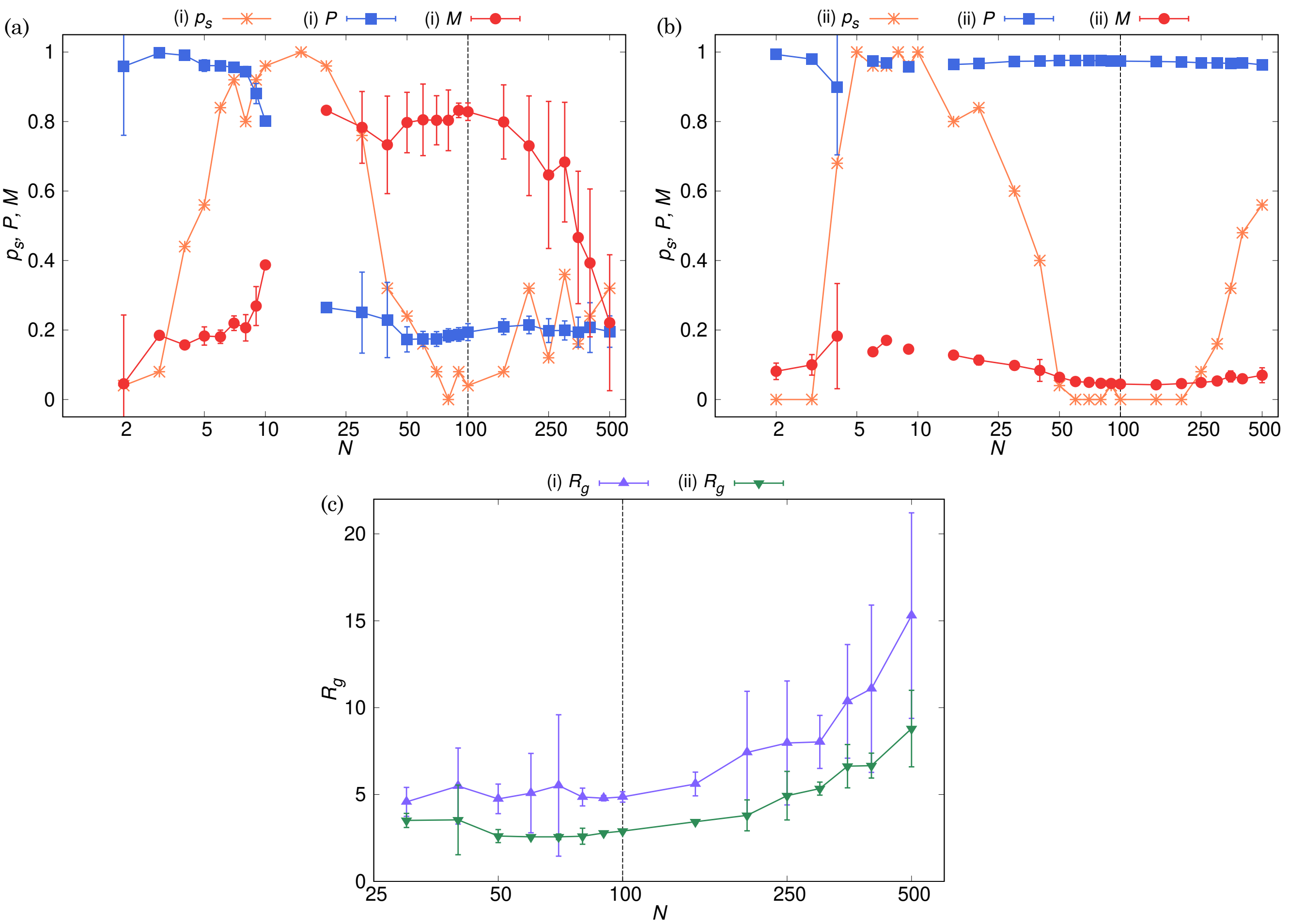

FIG. S15. The N dependence of (i) the vortex ($\chi = 0.3, \omega_o = 1.0$) and (ii) polarized school ($\chi = 0, \omega_o = 3.0$). (a),(b) p_s, P, and M as functions of N are represented in the same way as in Fig. S14(a)-(c). The dashed black vertical lines represent $N = 100$. (c) The radius of gyration R_g is plotted as a function of N by (i) purple and (ii) green solid lines.

IV. VISUAL INFORMATION AND TOPOLOGICAL DISTANCE

A. Occupancy ratio and distance to neighbors

The ratio of the image occupying the field of view is defined as

$$p_v(t) = \frac{1}{N}\sum_{i=1}^{N}\left(\frac{1}{N_b}\sum_{\mu=1}^{N_b}\delta_{i,\mu}(t)\right), \tag{S23}$$

where N_b is the total number of visual bins, and $\delta_{i,\mu}$ equals to 1 if the μ-th bin of the agent i is occupied by an image and 0 otherwise. The average distance from the eye to images is defined as

$$d_v(t) = \frac{1}{N}\sum_{i=1}^{N}\left(\frac{\sum_{\mu=1}^{N_b}\delta_{i,\mu}(t)r_{i,\mu}(t)}{\sum_{\mu=1}^{N_b}\delta_{i,\mu}(t)}\right), \tag{S24}$$

where $r_{i,\mu}$ is the distance of the neighbor in the μ-th bin (see Eq. (8) in the main text). In addition, we measure the minimum distance to neighbors

$$d_{\min}(t) = \frac{1}{N}\sum_{i=1}^{N}\min_{\mu=1,\cdots,N_b} r_{i,\mu}(t). \tag{S25}$$

We averaged $p_v(t)$, $d_v(t)$ and $d_{\min}(t)$ over the time window $t \in [300, 800]$ and over no-splitting simulations among 25 simulations as we mentioned in the previous section, to obtain p_v, d_v and $d_{\min}$ used in the main text. For random-random and random-aligned cases with 100 virtual agents (see the main text for the definition), the averages are taken over the same number of trial steps as the time steps for collective motion of real agents.

B. Functions of the topological distance

The occupancy of the image of the n-th nearest neighbor (nNN) p_n is defined as the percentage of the number of bins occupied by the image of nNN in all the bins occupied by the images:

$$p_n(t) = \frac{1}{N}\sum_{i=1}^{N}\left(\frac{\sum_{\mu=1}^{N_b}\delta_{i,\mu}^{(n)}(t)}{\sum_{\mu=1}^{N_b}\delta_{i,\mu}(t)}\right), \tag{S26}$$

where $\delta_{i,\mu}^{(n)}$ equals to 1 if the μ-th bin of the agent i is occupied by the image of nNN and is 0 otherwise. We averaged p_n over $t \in [300, 1300]$ in each simulation. As shown in Fig. S16(a), p_n shows a power-law behavior for small topological distances ($n \lesssim 10$), including the virtual agents cases. On the other hand, for large topological distances $n \gtrsim 30$, p_n shows an exponential behavior (Fig. S16(b)). We also calculate the cumulated occupancy $\sum_{m=1}^{n} p_m$. As shown in Fig. S16(c), it rapidly converges to 1 for (i)-(iii), but only slowly reaches 1 for the virtual agents cases. In addition, we measure the probability $P_{\phi,n}$ for the visual attention to be toward nNN, as the probability that the angle of the visual attention of an agent is within the bins occupied by the image of nNN in a simulation with $t \in [300, 1300]$. In other words, if $P_{\phi,n} = 1$, the visual attention is directed only to the n-th nearest neighbor. As shown in Fig. S16(d), the probabilities $P_{\phi,n}$ rapidly decays to zero as a function of n. We also define the probability that the visual attention is directed toward no neighbor, $P_{\phi,\mathrm{no}} = 1 - \sum_{n=1}^{N-1} P_{\phi,n}$. For a swarm, it is larger than $P_{\phi,\mathrm{no}}$ of a vortex and a polarized school, or $P_{\phi,1}$ of the swarm.

Finally, we measure the contribution of nNN in the interaction with neighbors. We define F_n and Ω_n as the absolute values of the speeding force $|F|$ and the angular velocity $|\Omega|$ in Eqs. (5),(6) in the main text, respectively, which are averaged over the time when the angle of the visual attention is within the bins occupied by the image of nNN. We also measured the expected values $\langle F_n\rangle = F_n P_{\phi,n}$ and $\langle \Omega_n\rangle = \Omega_n P_{\phi,n}$. In other words, F_n and Ω_n correspond to the instantaneous interaction when the visual attention of an agent is directed to nNN, and $\langle F_n\rangle$, $\langle \Omega_n\rangle$ correspond to the

averaged interaction during the simulation. We calculated F_n, Ω_n, $\langle F_n \rangle$, and $\langle \Omega_n \rangle$ in the time window $t \in [300, 800]$ using no-splitting cases in 25 simulations. As shown in Fig. S17(a), F_n is repulsive for topologically near neighbors (small n) and attractive for distant neighbors (large n), based on the formula Eq. (12). The peak of the attractive force shifts to large n as the cluster pattern changes from (iii) swarm to (i) vortex and (ii) polarized school, because the distance to neighbor d_v decreases from (iii) to (i) and (ii) (see Fig. 4(a)). On the other hand, $\langle F_n \rangle$ shows that the contribution from small n in the repulsive region is large as shown in Fig. S17(c). It indicates that the contribution of the attraction is screened in the cluster, while the repulsive contribution remains. Regarding Ω_n (see Fig. S17(b)), attraction mainly emerges based on Eq. (15), and $\langle \Omega_n \rangle$ is roughly equally contributed by several nearest neighbors (see Fig. S17(d)).

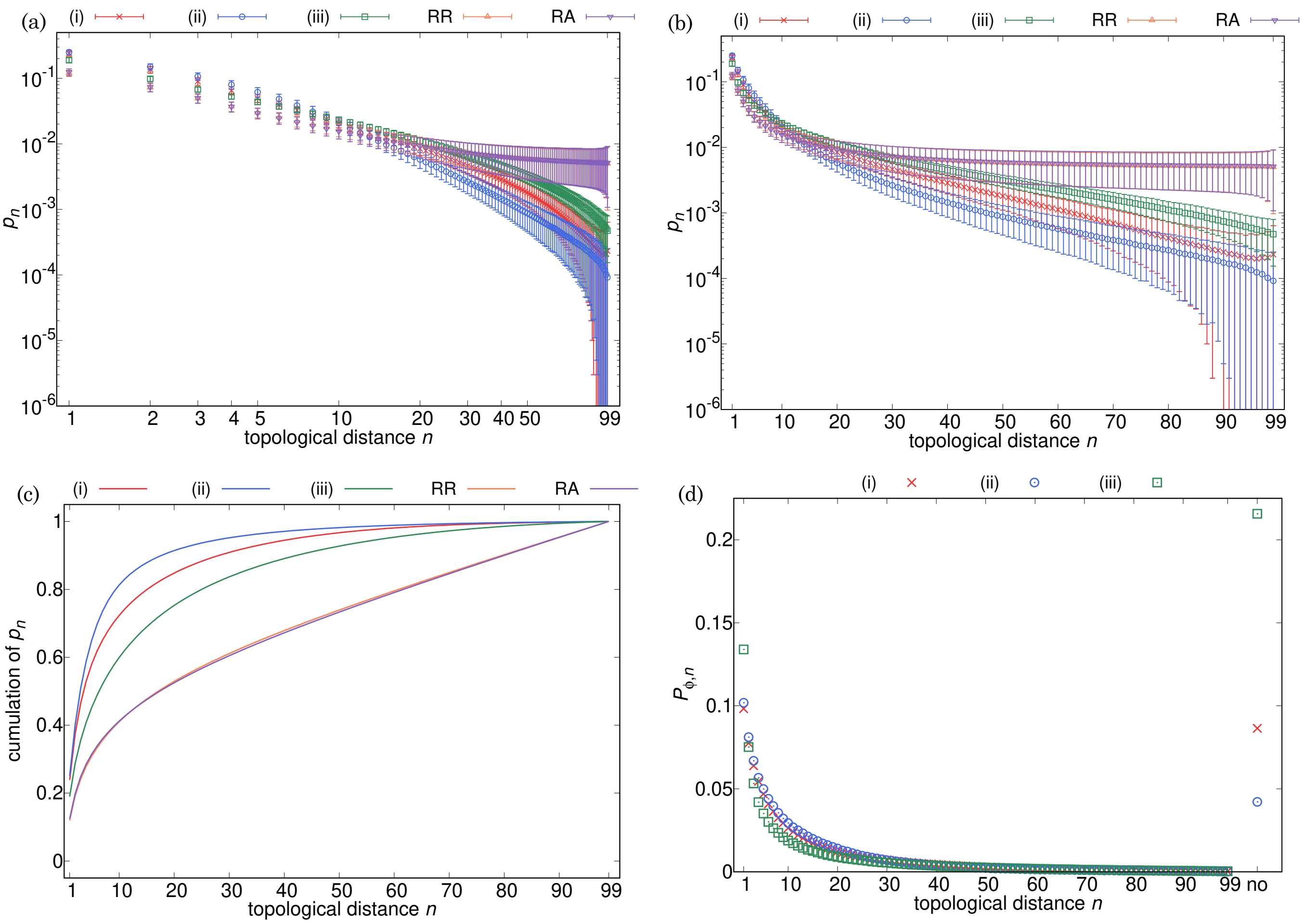

FIG. S16. Visual information of an agent in a cluster with 100 agents. The red, blue, green, and cyan correspond to the collective patterns (i)-(iv), and R-R (orange) and R-A (purple) correspond to the random-random case and the random-aligned case, respectively (see also the caption of Fig. 4). The occupancy p_n of the image of nNN in the (a) log-log and (b) semi-log plots. The errorbar represents the standard deviation. (c) The cumulated probability $\sum_{m=1}^{n} p_m$. (d) The probability $P_{\phi,n}$ of visual attention toward nNN; "no" on the horizontal axis represents that the visual attention is directed toward no neighbor.

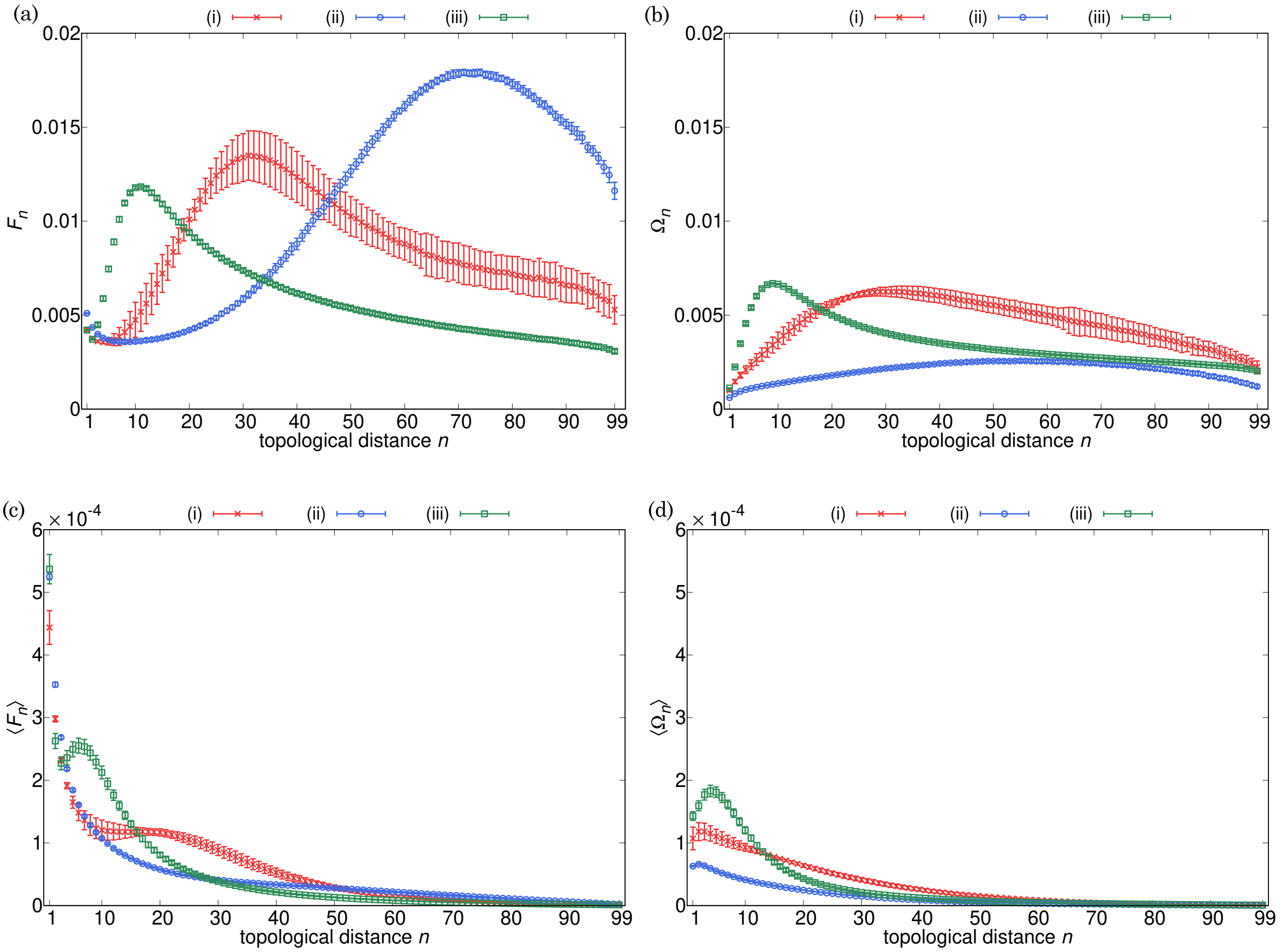

FIG. S17. Dependence of the interactions on the topological distance n. (a) F_n, (b) Ω_n, (c) $\langle F_n \rangle$, and (d) $\langle \Omega_n \rangle$. The red, blue, and green correspond to the collective patterns (i)-(iii), respectively. The errorbar represents the standard deviation.

V. FORCE MAP FOR PAIRWISE AND THREE-BODY INTERACTIONS

In this section, we use $(\chi, \omega_o) = (0.3, 1.0)$ for which a vortex emerges in the collective motion.

A. Definitions of the positional distribution and forces

For the initial condition for both two and three agents, the agents are randomly positioned in a circle of radius 7, and have random orientation, the speed $v_i = v_0$, and random angle of visual attention. The time window of a simulation is $t \in [0, 50]$, and we iterate 10000 simulations for the two agents case and 50000 simulations for the three agents case to obtain the maps. In the maps, the spacial range is $[-5, 5]$ with the bin width 0.1, the range of the relative speed is $[0, 3]$ with the bin width 0.1, and the range of the relative heading angle is $[-180°, 180°]$ with the bin width 2°. The relative position of a neighbor is measured between the centers of the body of a focal agent and the body of a neighbor, as shown in Fig. S18(a). The position of the center of the body of an agent is defined as $\boldsymbol{r}_i - l_e \boldsymbol{e}_i$.

The number density is obtained by counting the events in which a neighbor is located at a spatial bin, and then is normalized over the simulations. The speeding force is defined numerically as the component of the acceleration in the direction of motion,

$$\frac{\boldsymbol{v}_i(t) - \boldsymbol{v}_i(t - \Delta t)}{\Delta t} \cdot \boldsymbol{e}_i(t - \Delta t) \tag{S27}$$

(note that the body mass of an agent is 1). The turning force is the component perpendicular to the speeding force (see Fig. S18(a)),

$$\frac{\boldsymbol{v}_i(t) - \boldsymbol{v}_i(t - \Delta t)}{\Delta t} \cdot R_{-\frac{\pi}{2}} \boldsymbol{e}_i(t - \Delta t), \quad R_{-\frac{\pi}{2}} = \begin{bmatrix} 0 & 1 \\ -1 & 0 \end{bmatrix}. \tag{S28}$$

We then obtain the maps of the speeding and turning forces by averaging over a spatial bin, a relative speed bin, and a relative heading bin and over all the agents and simulations.

B. Maps for two agents

First, we show the results for two agents. As mentioned in the main text, the number density in the front is larger than in the rear (see Fig. 5(b)). If the neighbor is in the rear side, the orientation of the focal agent fluctuates due to the attraction from the rear, and therefore the relative position of the rear neighbor is widely distributed. Fig. S18(b),(c) show the maps of the speeding force. Regarding the relative speed dependence, the speeding attraction increases as the relative speed increases (see Fig. S18(b)). As shown in Fig. S18(c), backward attraction increases as the relative heading angle increases, while forward attraction is strong only in the range $[-90°, 90°]$, which results in avoiding collision. The speeding force has the reverse sign for $[-180°, -90°]$ and $[90°, 180°]$ compared to $[-90°, 90°]$. Fig. S18(d),(e) show the maps of the turning force. The turning attraction increases as the relative speed increases similarly to the speeding force (see Fig. S18(d)). As shown in Fig. S18(e), the turning attraction increases in particular when the neighbor is moving away, at the left with a positive relative heading angle or at the right with a negative relative heading angle.

C. Maps for three agents

Next, we show the results for three agents. As shown in Fig. S19(a), the number density is larger in the front than in the rear similarly to the case of two agents, but peaks of the number density appear also in the diagonally backward positions. We decompose the measured quantities as a function of the front-back position of neighbor 1 and neighbor 2, and a function of the left-right position of neighbor 1 and neighbor 2 (see Fig. S19(b),(c)). See the main text and

Fig. 5(e),(f) for the relation between the front-back position of neighbor 1 and neighbor 2 and the speeding force. As shown in Fig. S19(f), the number density as a function of the left-right position is extended in a relatively narrow region compared to the one as a function of the front-back position (Fig. 5(e)). The peak of the number density as a function of the left-right position is at the center of the map, which means that the neighbors take the position with nearly zero left-right distance. Combining with Fig. 5(e), we can conclude that three agents tend to take a linear configuration with the front-back distance of about 2.

As shown in Fig. S19(g)(h), the turning force of the three-body interaction is similar to the turning force of the averaged pairwise interaction. The averaged pairwise turning force is defined as $0.5 \times (F^{\text{pairwise}}_{\text{turning},1} + F^{\text{pairwise}}_{\text{turning},2})$, where $F^{\text{pairwise}}_{\text{turning},1}$ and $F^{\text{pairwise}}_{\text{turning},2}$ are the turning forces from the neighbor 1 and the neighbor 2 based on the assumption that the interaction between an agent and the neighbors is the pairwise interaction for two agents. (A similar definition is used for the averaged pairwise speeding force in the main text.) We find that the difference turning force is relatively small compared to the difference speeding force (see Fig. S19(i)).

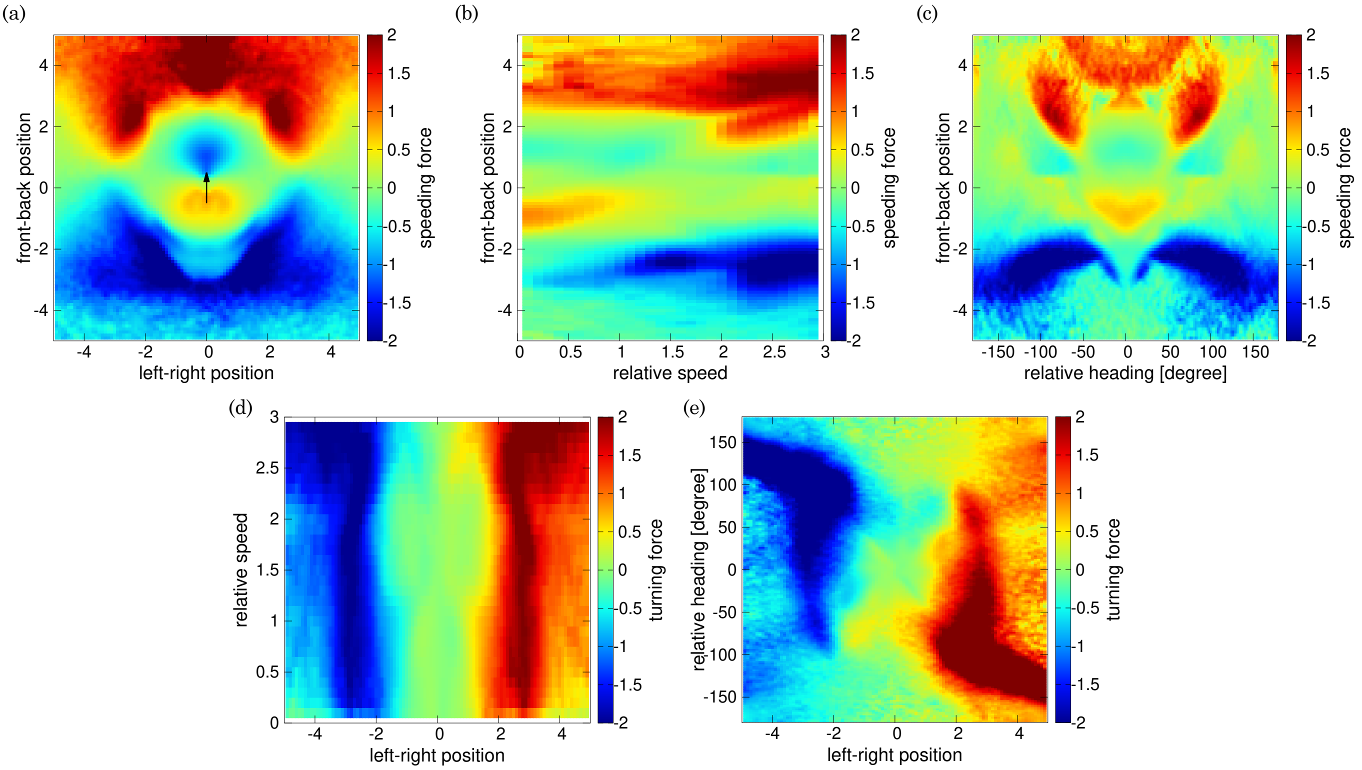

FIG. S18. Maps for two agents. The speeding force as functions of (a) the front-back and left-right positions of the neighbor, (b) the front-back position and relative speed, (c) the front-back position and relative heading. In (a), the self-propelling force is subtracted from the speeding force, and the black arrow in the center of the map represents the focal agent. The turning force as functions of (d) the front-back position and relative speed, (e) the front-back position and relative heading.

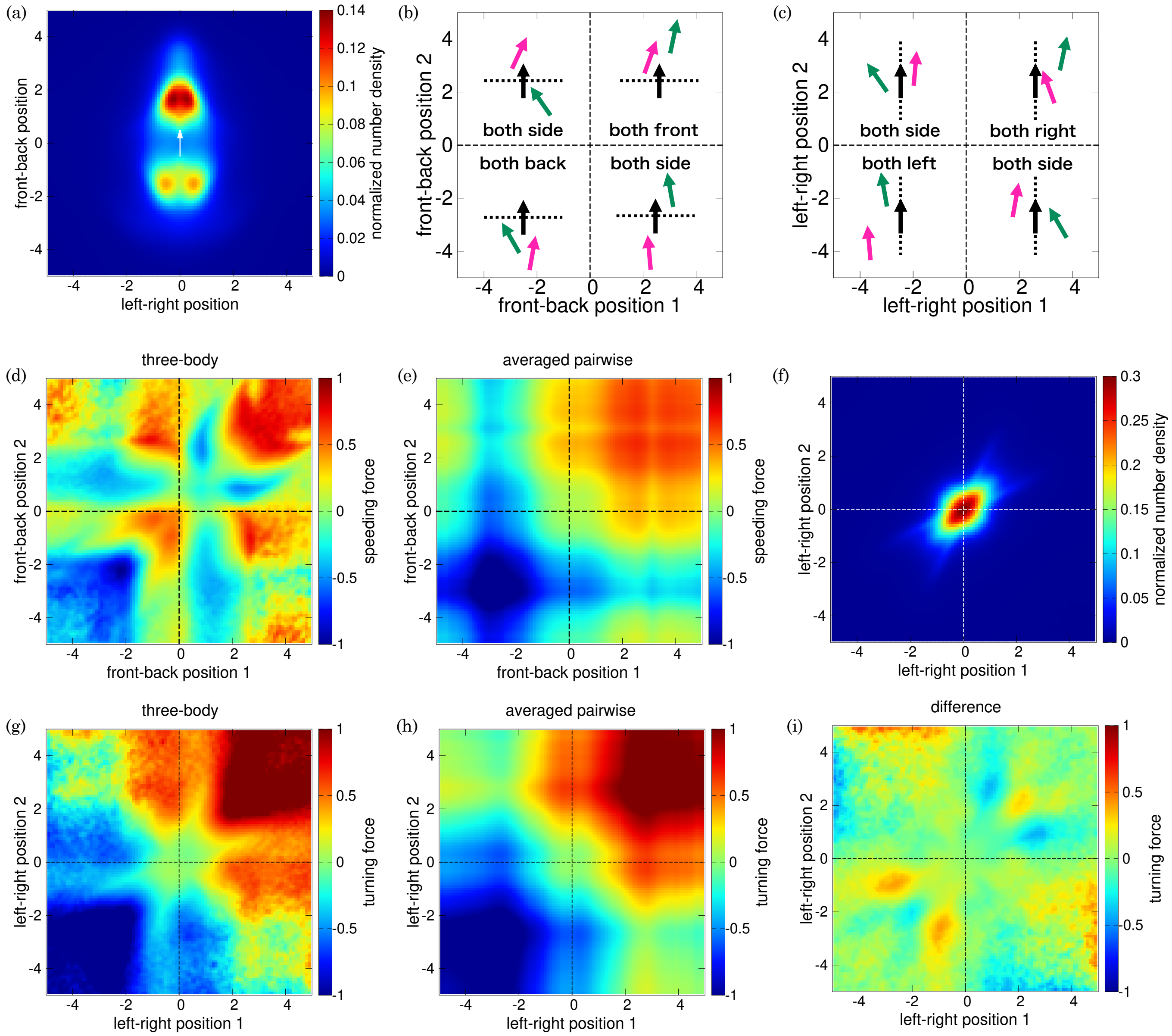

FIG. S19. Maps for three agents. (a) The normalized number density as a function of the position of neighbors. The white arrow in the center of the map represents the focal agent. In (b) and (c), we show schematic pictures of the relation between a focal agent (black arrow), the neighbor 1 (green arrow) and neighbor 2 (pink arrow) with respect to (b) the front-back positions and (c) the left-right positions. (d) The three-body speeding force and (e) the averaged pairwise speeding force. (f) The normalized number density as a function of the left-right position. (g) The three-body turning force, (h) the averaged pairwise turning force, and (i) the difference turning force.

VI. SUPPLEMENTAL MOVIES

The coordinate system of the movies is the center-of-mass system. The agents are represented in the same way as in Fig. 3(a). In the movies, χ and ω_o are denoted as chi and omegao, respectively. The movies start at $t = 0$ unless otherwise stated.

- **Movie. S1**: (i) A vortex for $\chi = 0.3, \omega_o = 1.0$.
- **Movie. S2**: (ii) A polarized school for $\chi = 0, \omega_o = 3.0$.
- **Movie. S3**: (iii) A swarm for $\chi = 0.5, \omega_o = 0.25$.
- **Movie. S4**: (iv) A turning cluster for $\chi = 1.0, \omega_o = 3.0$.
- **Movie. S5**: (v) Unsteady aggregation for $\chi = 0.6, \omega_o = 1.75$. The movie starts at $t = 250$.

---

[1] H. Kunz and C. K. Hemelrijk, Appl. Anim. Behav. Sci. **138**, 142 (2012).
[2] D. J. G. Pearce, A. M. Miller, G. Rowlands, and M. S. Turner, Proc. Natl. Acad. Sci. U.S.A. **111**, 10423 (2014).
[3] B. H. Lemasson, J. J. Anderson, and R. A. Goodwin, J. Theor. Biol. **261**, 501 (2009).
[4] B. H. Lemasson, J. J. Anderson, and R. A. Goodwin, Proc. R. Soc. B **280**, 20122003 (2013).
[5] R. Bastien and P. A. Romanczuk, Sci. Adv. **6**, eaay0792 (2020).
[6] J. Qi, L. Bai, Y. Wei, H. Zhang, and Y. Xiao, IEEE Internet Things J. **10**, 10368 (2023).
[7] D. Castro, F. Ruffier, and C. Eloy, Phys. Rev. Research **6**, 023016 (2023).
[8] B. Collignon, A. Séguret , and J. Halloy, R. Soc. open sci. **3**, 150473 (2016).
[9] V. H. Sridhar, L. Li, D. Gorbonos, M. Nagy, B. R. Schell, T. Sorochkin, N. S. Gov, and I. D. Couzin, Proc. Natl. Acad. Sci. U.S.A. **118**, e2102157118 (2021).
[10] D. Gorbonos, N. S. Gov, and I. D. Couzin, PRX Life **2**, 013008 (2024).
[11] L. Oscar, L. Li, D. Gorbonos, I. D. Couzin, and N. S. Gov, Phys. Biol. **20**, 045002 (2023).
[12] R. Harpaz, M. N. Nguyen, A. Bahl, and F. Engert, Nat. Commun. **12**, 6578 (2021).
[13] A. W. Hein, Curr. Opin. Neurobiol. **74**, 102551 (2022).
[14] V. H. Sridhar, J. D. Davidson, C. R. Twomey, M. M. G. Sosna, M. Nagy, and I. D. Couzin, Phil. Trans. R. Soc. B **378**, 20220062 (2023).
[15] D. Pita, B. A. Moore, L. P. Tyrrell, and E. Fernández-Juricic, PeerJ **3**, e1113 (2015).
[16] A. Kabayama, G. Kawamura, and T. Yonemori, Bull. Jpn. Soc. Sci. Fish. **45**, 1481 (1979).
[17] A. Filella, F. Nadal, C. Sire, E. Kanso, and C. Eloy, Phys. Rev. Lett. **120**, 198101 (2018).
[18] S. Ito and N. Uchida, J. Phys. Soc. Jpn. **91**, 064806 (2022).
[19] M. Gazzola, M. Argentina, and L. Mahadevan, Nat. Phys. **10**, 758 (2014).
[20] Y. Katz, K. Tunstrøm, C. C. Ioannou, C. Huepe, and I. D. Couzin, Proc. Natl. Acad. Sci. U.S.A. **108**, 18720 (2011).
[21] J. E. Herbert-Read, A. Perna, R. P. Mann, T. M. Schaerf, D. J. T. Sumpter, and A. J. W. Ward, Proc. Natl. Acad. Sci. U.S.A. **108**, 18726 (2011).
[22] D. S. Calovi, A. Litchinko, V. Lecheval, U. Lopez, A. P. Escudero, H. Chaté, C. Sire, and G. Theraulaz, PLOS Comput. Biol. **14**, e1005933 (2018).
[23] R. E. Jones, R. J. Petrell, and D. Pauly, Aquac. Eng. **20**, 216 (1999).
[24] T. Y. Wu, in *Scale Effects in Animal Locomotion*, edited by T.J. Pedley (Academic Press, London/New York, 1977), p. 203.
[25] J. J. Videler and C. S. Wardle, Rev. Fish Biol. Fish **1**, 23 (1991).
[26] G. V. Lauder and E. D. Tytell, Fish Physiology **23**, 425 (2005).
[27] E. D. Tytell, Proc. R. Soc. Lond. B **271**, 2535 (2004).
[28] T. N. Wise, M. A. B. Schwalbe, and E. D. Tytell, J. Exp. Biol. **221**, jeb190892 (2018).
[29] T. Tandler, E. Gellman, D. De La Cruz, and D. J. Ellerby, J. Fish Biol. **94**, 532 (2019).
