# Supplementary figures and images for: Selective decision-making and collective behavior of fish by the motion of visual attention
Source: PNAS Nexus. 2024 Jul 2;3(7):pgae264. doi: 10.1093/pnasnexus/pgae264 (PMC11264410; doi:10.1093/pnasnexus/pgae264)

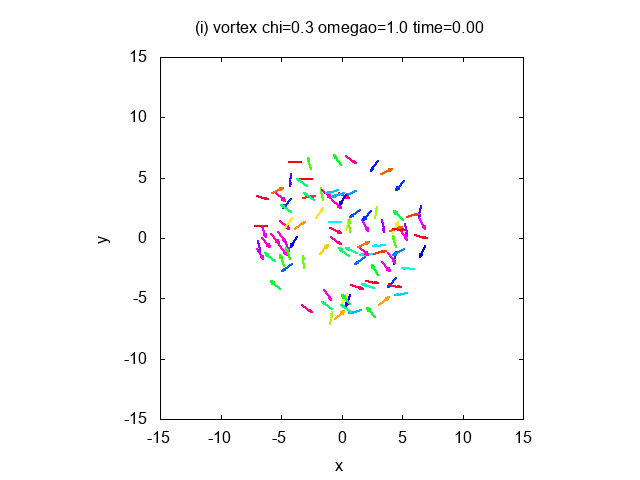

Supplement: pgae264_Supplementary_Data [file pgae264_supplementary_data.zip › PNASNEXUS-PNASNEXUS-2024-00298-TR-s02.gif]

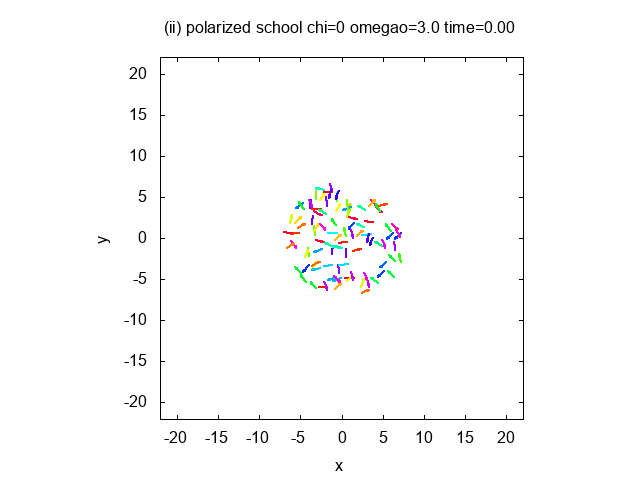

Supplement: pgae264_Supplementary_Data [file pgae264_supplementary_data.zip › PNASNEXUS-PNASNEXUS-2024-00298-TR-s03.gif]

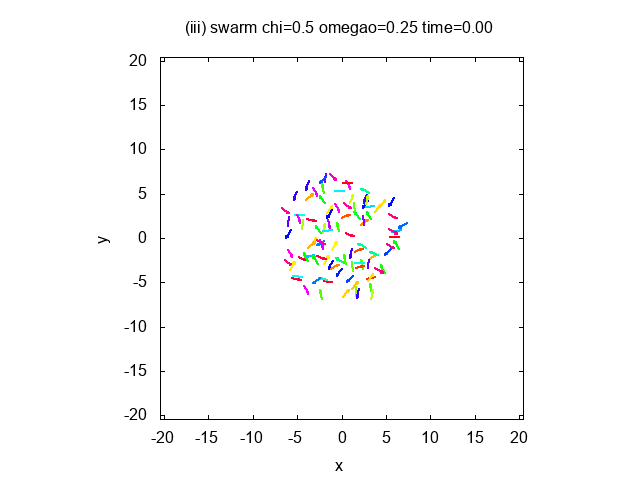

Supplement: pgae264_Supplementary_Data [file pgae264_supplementary_data.zip › PNASNEXUS-PNASNEXUS-2024-00298-TR-s04.gif]

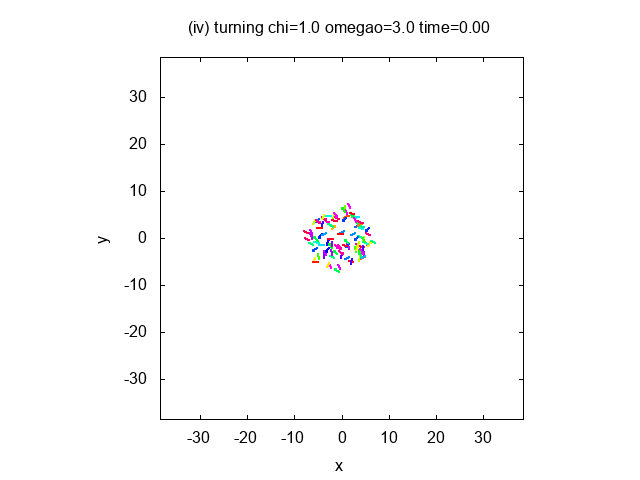

Supplement: pgae264_Supplementary_Data [file pgae264_supplementary_data.zip › PNASNEXUS-PNASNEXUS-2024-00298-TR-s05.gif]

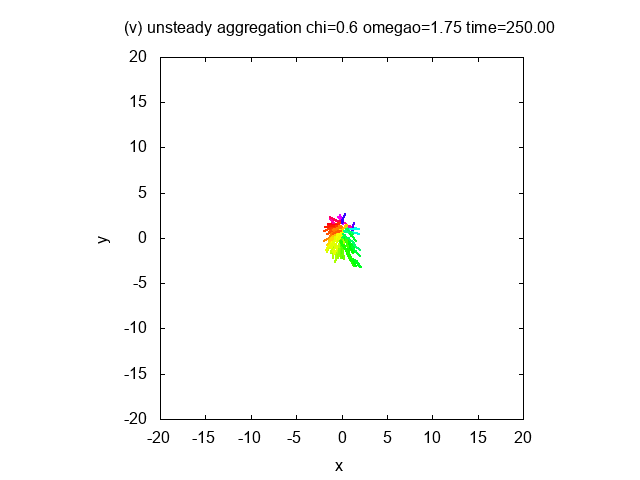

Supplement: pgae264_Supplementary_Data [file pgae264_supplementary_data.zip › PNASNEXUS-PNASNEXUS-2024-00298-TR-s06.gif]
